# Two VQ Proteins are Substrates of the OsMPKK6-OsMPK4 Cascade in Rice Defense Against Bacterial Blight

**DOI:** 10.1186/s12284-021-00483-y

**Published:** 2021-04-28

**Authors:** Na Li, Zeyu Yang, Juan Li, Wenya Xie, Xiaofeng Qin, Yuanrong Kang, Qinglu Zhang, Xianghua Li, Jinghua Xiao, Haigang Ma, Shiping Wang

**Affiliations:** National Key Laboratory of Crop Genetic Improvement, National Center of Plant Gene Research (Wuhan), Huazhong Agricultural University, Wuhan, 430070 China

**Keywords:** VQ protein, MAPK, Bacterial blight, Phosphorylation, *Oryza sativa*

## Abstract

**Background:**

The plant-specific valine-glutamine (VQ) protein family with the conserved motif FxxxVQxLTG reportedly functions with the mitogen-activated protein kinase (MAPK) in plant immunity. However, the roles of VQ proteins in MAPK-mediated resistance to disease in rice remain largely unknown.

**Results:**

In this study, two rice VQ proteins OsVQ14 and OsVQ32 were newly identified to function as the signaling components of a MAPK cascade, OsMPKK6-OsMPK4, to regulate rice resistance to *Xanthomonas oryzae* pv. *oryzae* (*Xoo*). Both *OsVQ14* and *OsVQ32* positively regulated rice resistance to *Xoo*. In vitro and in vivo studies revealed that OsVQ14 and OsVQ32 physically interacted with and were phosphorylated by OsMPK4. OsMPK4 was highly phosphorylated in transgenic plants overexpressing *OsMPKK6*, which showed enhanced resistance to *Xoo*. Meanwhile, phosphorylated OsVQ14 and OsVQ32 were also markedly accumulated in *OsMPKK6-*overexpressing transgenic plants.

**Conclusions:**

We discovered that OsVQ14 and OsVQ32 functioned as substrates of the OsMPKK6-OsMPK4 cascade to enhance rice resistance to *Xoo*, thereby defining a more complete signal transduction pathway for induced defenses.

**Supplementary Information:**

The online version contains supplementary material available at 10.1186/s12284-021-00483-y.

## Background

Rice bacterial blight, caused by *Xanthomonas oryzae* pv. *oryzae* (*Xoo*), is one the most serious diseases affecting rice (*Oryza sativa*) worldwide, resulting in significant damages in rice quality and yield (Nino-Liu et al. 2006; Jiang et al. 2020). Development of host plant immunity has been considered as one of the best choices available for achieving economical and sustainable management of bacterial blight. Rice resistance to *Xoo* is mediated by major disease resistance (*MR*) genes and quantitative trait loci (QTLs) (Kou and Wang 2010; Zhang and Wang 2013). Molecular characterization of these *Xoo*-resistance genes and related pathways are therefore essential for development of broad-spectrum, durable resistance.

The mitogen-activated protein kinase (MAPK) cascade is composed of a MAPK kinase kinase (MAPKKK), a MAPK kinase (MAPKK) and a MAPK, and regulates plant defense response through sequential phosphorylation (Zhang et al. 2018). MAPKKK phosphorylates MAPKK, thereby leading to MAPK phosphorylation within the conserved Thr-X-Tyr activation motif. Several MAPK cascades have been identified in rice response to pathogen infection. OsMPKKKε-OsMPKK4/5-OsMPK3/6 cascade regulates chitin signaling in rice resistance to the blast fungus *Magnaporthe oryzae* (Wang et al. 2017). OsMPKKK11 and OsMPKKK18 also activate OsMPKK4-OsMPK3/6 cascade in chitin signal transduction (Yamada et al. 2017). OsMPKK10.2-OsMPK6 cascade promotes rice resistance to *M. oryzae* and *X. oryzae* pv. *oryzicola* via the activation of salicylic acid (SA) transduction (Ueno et al. 2015; Ma et al. 2017, 2021). Additionally, several MAPKKK and MAPK genes have been identified in regulating resistance to diseases in rice. *OsEDR1* (*OsMPKKK1*) negatively regulates resistance to *Xoo* but elevates resistance to *M. oryzae* via the activation of ethylene synthesis (Shen et al. 2011). *OsMPK4* (the ortholog of *Arabidopsis MPK4*) and *OsMPK17–1* (the ortholog of *Arabidopsis MPK17*) contribute to resistance to *Xoo* infection (Shen et al. 2010; Seo et al. 2011). *OsMPK15* negatively regulates resistance to *M. oryzae* and *Xoo* (Hong et al. 2019). Furthermore, all the MAPKKK, MAPKK and MAPK genes involved in rice–*Xoo* interactions have been extensively analyzed (Yang et al. 2015).

Valine-glutamine (VQ) motif containing proteins, a class of plant-specific protein with the conserved FxxxVQxLTG amino acid sequence (where “x” represents any amino acid) and thus being termed the VQ-protein family, play key roles in the defense signal transduction process in plants (Yuan et al. 2021). AtSIB1 (AtVQ23) and AtSIB2 (AtVQ16) positively regulate defenses against necrotrophic pathogens via interaction with AtWRKY33 in *Arabidopsis* (Lai et al. 2011). AtVQ10 positively regulates *Arabidopsis* resistance to *Botrytis cinerea* via interaction with AtWRKY8 (Chen et al. 2018)*.* JAV1 (AtVQ22), which forms a complex with JAZ8-WRKY51 to repress jasmonic acid (JA) biosynthesis, is rapidly phosphorylated in a Ca^2+^/calmodulin-dependent manner after injury caused by insect herbivory, and in turn dissolves the interaction with JAZ8-WRKY51 to activate JA biosynthesis for plant defenses (Hu et al. 2013; Yan et al. 2018). *OsVQ13* promotes rice resistance to *Xoo* by activating the OsMPK6-OsWRKY45 signaling pathway (Uji et al. 2019).

Research in *Arabidopsis* has revealed that VQ proteins are phosphorylated by the MAPKs to regulate plant defense responses. AtMKS1 (AtVQ21) is phosphorylated by AtMPK4 that is activated by pathogen infection, and subsequently releases from the AtMPK4-AtMKS1-AtWRKY33 complex to induce the expression of *PHYTOALEXIN DEFICIENT 3*, thereby resulting in the elevated resistance to disease (Andreasson et al. 2005; Qiu et al. 2008; Petersen et al. 2010). At least 10 VQ proteins (AtVQ4, AtVQ6, AtVQ9, AtVQ11, AtVQ13, AtVQ14, AtVQ19, AtVQ31, AtVQ32, AtVQ33) have been observed to be phosphorylated by AtMPK3/AtMPK6 (Pecher et al. 2014); however, the biological function of the phosphorylation remained largely unknown. The rice genome contains 40 VQ genes (Li et al. 2014a). So far, only *OsVQ13* has been observed to mediate the biological process of rice defense responses (Uji et al. 2019). The MAPK-dependent regulatory mechanisms underlying the function of VQ proteins require further investigation.

In the present study, we characterized the functions of two VQ proteins, OsVQ14 and OsVQ32 (the homologs of AtVQ21) in rice defense responses. Our investigation confirmed positive regulation of both *OsVQ14* and *OsVQ32* in rice resistance to *Xoo*. In vitro and in vivo tests further revealed that OsVQ14 and OsVQ32 functioned as phosphorylation substrates of OsMPK4 to physically interact with OsMPK4. In vivo phosphorylation assays revealed that OsMPK4 was highly phosphorylated in the *OsMPKK6-*overexpressing transgenic lines. Furthermore, the phosphorylated OsVQ14 and OsVQ32 were accumulated in *OsMPKK6*-overexpressing transgenic plants, resulting in the elevated resistance to *Xoo*. The results demonstrated that OsVQ14 and OsVQ32 functioned as the substrates of the OsMPKK6-OsMPK4 cascade to enhance rice resistance to *Xoo*.

## Results

### *OsVQ14* and *OsVQ32* Positively Regulate Rice Resistance to *Xoo*

**Fig. 1:**
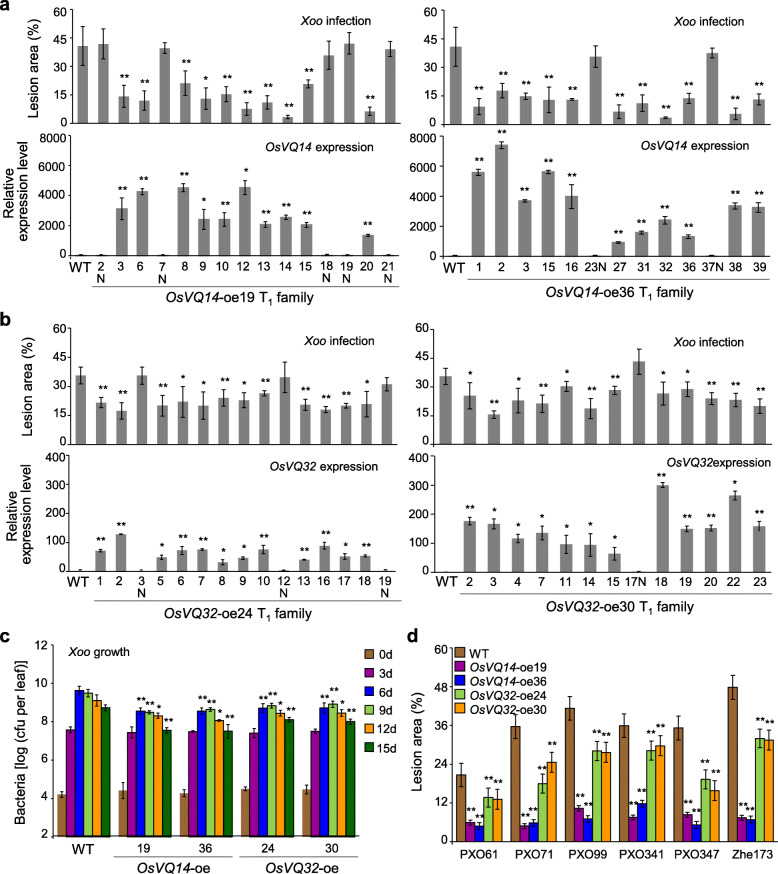
Overexpressing *OsVQ14* and *OsVQ32* enhanced rice resistance to *Xoo*. The asterisks “**” or “*” indicate a significant difference between transgenic plants and wild type (WT; Zhonghua 11) plants at *P* < 0.01 or *P* < 0.05, respectively. N, negative siblings segregated from the T_1_ families. **a** and **b** The enhanced resistance of the transgenic plants to *Xoo* is associated with *OsVQ14* expression (**a**) and *OsVQ32* expression (**b**) in two T_1_ families. Bars represent mean (3 to 5 leaves from one plant for lesion area, and 3 replicates for expression level) ± standard deviation (SD). **c** Analysis of the *Xoo* growth in leaves of *OsVQ14*-oe and *OsVQ32*-oe plants. Bars represent mean (3 leaves from 3 positive plants) ± SD. The significant difference was detected between transgenic plants and WT with the same treatment. cfu, colony-forming unit. **d** Analysis of the response of *OsVQ14*-oe and *OsVQ32*-oe plants to different *Xoo* strains. Bars represent mean (3 plants, with each plant having 3 to 5 leaves for lesion area) ± SD

Our previous research revealed that *Xoo* infection strongly induced the expression of *OsVQ14* and *OsVQ32* in rice (Li et al. 2014a). Two genes *OsVQ14* and *OsVQ32* herein were separately overexpressed in the rice variety Zhonghua 11 (wild type, WT) to further investigate their biological functions. Positive transgenic plants with high *OsVQ14* or *OsVQ32* expression showed significantly enhanced resistance (*P* < 0.01) to *Xoo* strain PXO347, with the lesion areas ranging from 17.4% to 31.5% in *OsVQ14*-oe plants and 4.8% to 24.6% in *OsVQ32*-oe plants, compared to 35.5% in the WT (Fig. S1). T_1_ progenies derived from two *OsVQ14*-oe lines (19 and 36) and two *OsVQ32*-oe lines (24 and 30) were further examined with *Xoo.* Compared to the WT, the *OsVQ14*-oe plants and *OsVQ32*-oe plants exhibited the improved resistance to *Xoo* (Fig. 1a and b). The lesion areas were significantly correlated with the expression levels of *OsVQ14* or *OsVQ32*. The correlation coefficients were − 0.776 and − 0.547 (*n* = 15, *P* < 0.01 and *n* = 13, *P* < 0.05) in *OsVQ14*-oe19 and *OsVQ14*-oe36, and − 0.828 and − 0.553 (*n* = 15, *P* < 0.01 and *n* = 13, *P* < 0.05) in *OsVQ32*-oe24 and *OsVQ32*-oe30, respectively (Fig. 1a and b). The data suggested that the increased resistance was significantly correlated with elevated expression levels of *OsVQ14* and *OsVQ32*. Furthermore, compared to WT at 6 to 15 days after inoculation, the reduction of growth rates of *Xoo* in rice leaves were 7.6- to 16.7-fold in *OsVQ14*-oe plants and 3.8- to 8.2-fold in *OsVQ32*-oe plants (Fig. 1c).

*OsVQ14*-oe and *OsVQ32*-oe plants were further inoculated with five other *Xoo* strains (PXO61, PXO71, PXO99, PXO341, and Zhe173). As shown in Fig. 1d, the lesion areas of *OsVQ14*-oe and *OsVQ32*-oe plants were significantly reduced compared to the WT (*P* < 0.01), indicating that the *OsVQ14*-oe and *OsVQ32*-oe plants were significantly resistant to all five *Xoo* strains.

**Fig. 2:**
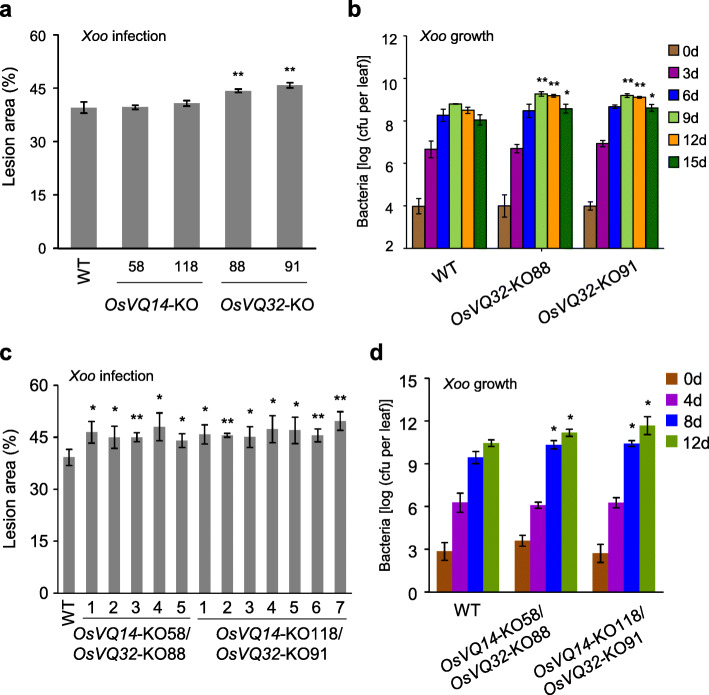
Knocking out *OsVQ32* reduced rice resistance to *Xoo*. The asterisks “**” or “*” indicate a significant difference between transgenic plants and wild type (WT; Zhonghua 11) at *P* < 0.01 or *P* < 0.05, respectively. **a** Analysis of the response of *OsVQ14*-KO and *OsVQ32*-KO plants to *Xoo* inoculation. Bars represent mean (3 to 5 plants, with each plant having 3 leaves for lesion area) ± standard deviation (SD). **b** Analysis of the *Xoo* growth in leaves of *OsVQ32*-KO plants. Bars represent mean (3 leaves from 3 positive plants) ± SD. The significant difference was detected between transgenic plants and WT with the same treatment. cfu, colony-forming unit. **c** Analysis of the response of *OsVQ14*-KO/*OsVQ32*-KO double mutants to *Xoo* inoculation. Bars represent mean (3 leaves from one plant for lesion area) ± standard deviation (SD). **d** Analysis of the *Xoo* growth in leaves of *OsVQ14*-KO/*OsVQ32*-KO double mutants. Bars represent mean (3 leaves from 3 positive plants) ± SD. The significant difference was detected between transgenic plants and WT with the same treatment. cfu, colony-forming unit

We further generated knock-out (KO) mutations of *OsVQ14* and *OsVQ32* in the WT using CRISPR/Cas9 (Clustered Regularly Interspaced Short Palindromic Repeats/CRISPR-associated protein 9). Two target sites were selected for each *VQ* gene (Fig. S2a and c). The T_1_ lines of two homozygous KO mutants for each *VQ* gene were inoculated with *Xoo* (Fig. S2b and d). As shown in Fig. 2a, the lesion areas in two homozygous *OsVQ14*-KO lines (58 and 118) were similar to those in the WT. However, the lesion areas in two homozygous *OsVQ32*-KO lines (88 and 91) were significantly greater than those in the WT (*P* < 0.01) (Fig. 2a). In addition, the *Xoo* growth rates in homozygous *OsVQ32*-KO plants were 1.6- to 5.0-fold higher than those in the WT at 9 to 15 days after inoculation (Fig. 2b). Next, we generated the *OsVQ14*-KO/*OsVQ32*-KO double mutant by crossing the *OsVQ14*-KO plants with the *OsVQ32*-KO plants. The *OsVQ14*-KO/*OsVQ32*-KO double mutant showed increased susceptibility to *Xoo*, resulting in significantly increased lesion area and *Xoo* growth compared with WT (*P* < 0.05) (Fig. 2c and d).

To confirm whether CRISPR/Cas9 caused off-target mutations in *OsVQ32*-KO plants, the genome-wide potential off-target sites were analyzed using the CRISPR-P website (http://cbi.hzau.edu.cn/crispr/) (Liu et al. 2017) (Table S1). The sequencing results verified that none of the potential off-target sites contained any DNA mutations (Fig. S3), indicating that the *OsVQ32*-KO phenotypes were unlikely contributed by off-target mutations in this study. Taken together, these results revealed that both *OsVQ14* and *OsVQ32* positively regulate rice resistance to *Xoo* infection.

### OsVQ14 and OsVQ32 Interact with OsMPK4

**Fig. 3:**
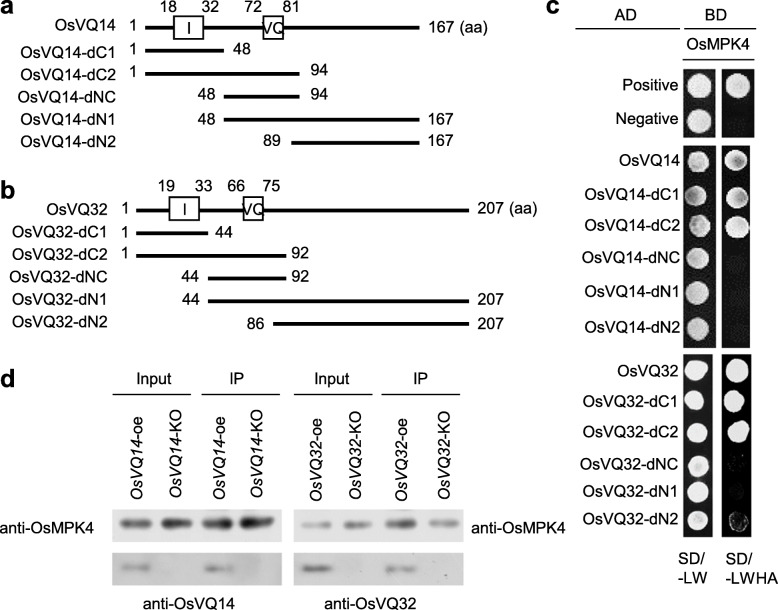
OsVQ14 and OsVQ32 interacted with OsMPK4. **a** and **b** Schematic representation of OsVQ14 truncations (**a**) and OsVQ32 truncations (**b**). Rectangles “I” and “VQ” mean domain I and VQ domain, respectively. **c** Interaction of OsMPK4 with OsVQ14 or OsVQ32 was analyzed by yeast two-hybrid assay. The interactions were assessed by growing yeast cells on synthetic defined premixes (SD) medium lacking (−) leucine (L), tryptophan (W), histidine (H), and adenine (A). Co-transformation of BD-53 and AD-RecT was used as positive control while co-transformation of BD-Lam and AD-RecT was used as negative control. AD, Activation domain; BD, DNA-binding domain. **d** Interaction of OsMPK4 with OsVQ14 or OsVQ32 in rice plants was analyzed by co-immunoprecipitation assay. Total protein was extracted from rice leaves and anti-OsMPK4 antibody was used for the immunoprecipitation (IP)

Previous studies demonstrated that AtVQ21 (AtMKS1) is the substrate of AtMPK4 in *Arabidopsis* (Andreasson et al. 2005; Qiu et al. 2008). Because OsVQ14 and OsVQ32 are homologs of AtVQ21 (Figs. S4 and S5) and OsMPK4 (the ortholog of *Arabidopsis* MPK4) is instrumental in rice resistance to *Xoo* infection (Shen et al. 2010), we performed yeast two-hybrid (Y2H) analysis to detect whether OsVQ14 and OsVQ32 interact with OsMPK4. The results showed that both OsVQ14 and OsVQ32 strongly interacted with OsMPK4 in yeast cells (Fig. 3a-c). To further specify which domains of OsVQ14 and OsVQ32 were involved in the interactions, we performed Y2H analysis using two N-terminal deletions, two C-terminal deletions, and a VQ domain (Fig. 3a and b). The data showed that the C-terminal deletions of OsVQ14 and OsVQ32, designated as OsVQ14(32)-dC1 and OsVQ14(32)-dC2 (both containing domain I that containing the putative MAPK docking domain), interacted with OsMPK4 (Fig. 3c). However, no interaction was observed between the N-terminal deletions (designated as OsVQ14(32)-dN1, OsVQ14-dN2, and OsVQ14(32)-dNC) and OsMPK4, and only a weak interaction was detected between OsVQ32-dN2 and OsMPK4 (Fig. 3c).

The interactions of OsVQ14 and OsVQ32 with OsMPK4 were further confirmed in rice plants. OsMPK4 was immunoprecipitated from the protein complex extracted from rice leaves, and OsVQ14 or OsVQ32 was detected in immunoprecipitated protein extracts obtained from *OsVQ14*-oe or *OsVQ32*-oe transgenic plants instead of *OsVQ14*-KO or *OsVQ32*-KO plants (Fig. 3d). These results suggested that OsMPK4 physically interacts with OsVQ14 and OsVQ32 in vivo.

### OsMPK4 Phosphorylates OsVQ14 and OsVQ32

**Fig. 4:**
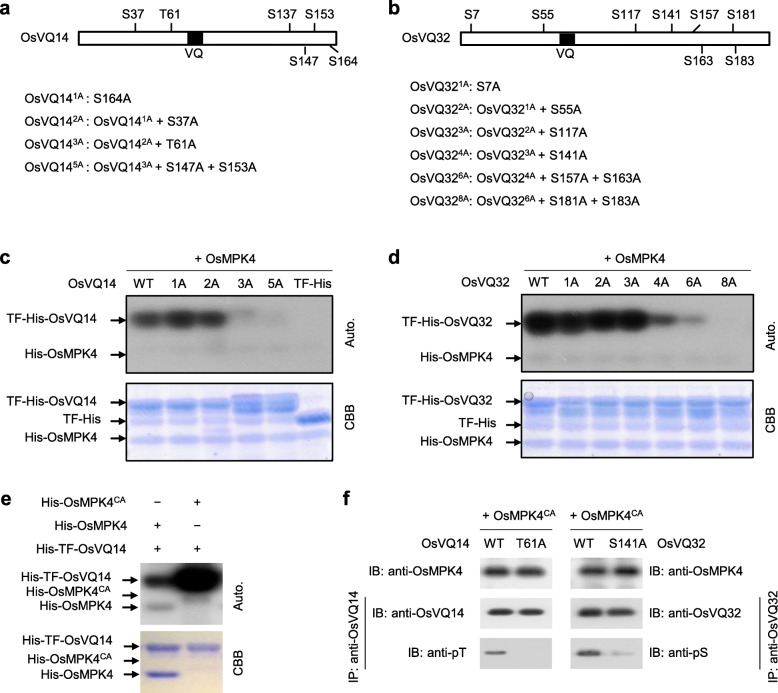
OsMPK4 phosphorylated OsVQ14 and OsVQ32. **a** and **b** Mutagenesis of putative MAPK phosphorylation sites of OsVQ14 (**a**) and OsVQ32 (**b**). **c** and **d** Phosphorylation assays of wild-type (WT) and mutated OsVQ14 (**c**) and OsVQ32 (**d**) by OsMPK4 in vitro. TF-His, trigger factor (TF) and histidine (His) tag. Auto., Autoradiograph; CBB, Coomassie brilliant blue staining. **e** In vitro kinase activity toward OsVQ14 of OsMPK4 and its constitutively active (CA) mutant. **f** Phosphorylation assays of wild-type (WT) and mutated OsVQ14 and OsVQ32 by OsMPK4^CA^ in vivo. IP, immunoprecipitated

MAPK primarily functions to recognize and phosphorylate target substrates on serine (S) or threonine (T) residues, followed by proline (Tanoue and Nishida 2003). Amino acid sequence analysis showed that OsVQ14 and OsVQ32 carried six potential MAPK phosphorylation sites (S37, T61, S137, S147, S153, and S164) and eight sites (S7, S55, S117, S141, S157, S163, S181, and S183) (Fig. 4a and b), respectively. The potential MAPK phosphorylation residues (S or T) were then cumulatively substituted with alanine (A) (Fig. 4a and b) for in vitro phosphorylation assays with the proteins purified from the bacterium. The results showed that trigger factor (TF)- and His-tagged OsVQ14 and OsVQ32 (TF-His-OsVQ14 and TF-His-OsVQ32) were strongly phosphorylated by His-tagged OsMPK4 (His-OsMPK4) (Fig. 4c and d). In OsVQ14, simultaneous substitutions of S37 and S164 (OsVQ14^2A^) did not affect the phosphorylation process, whereas further substitution of T61 (OsVQ14^3A^) abolished almost all phosphorylation. As S137 failed to be replaced by A, the other five residues were substituted to generate OsVQ14^5A^, in which all phosphorylation had already been abolished (Fig. 4c). In OsVQ32, simultaneous substitutions of S7, S55 and S117 (OsVQ32^3A^) did not affect the phosphorylation process, whereas further substitution of S141 (OsVQ32^4A^) abolished most phosphorylation, and the substitutions of all eight residues (OsVQ32^8A^) abolished phosphorylation entirely (Fig. 4d). These results suggest that OsMPK4 can phosphorylate OsVQ14 and OsVQ32, and that the T61 in OsVQ14 and S141 in OsVQ32 are essential for successful phosphorylation.

The constitutively active (CA) version of OsMPK4 (where aspartic acid at 198 and glutamic acid at 202 were replaced by glycine and alanine, respectively) (Berriri et al. 2012) was generated to determine OsMPK4-mediated OsVQ14 and OsVQ32 phosphorylation in vivo. In vitro phosphorylation assay showed that the His-OsMPK4^CA^ exhibited obviously increased kinase activity toward His-TF-OsVQ14 than His-OsMPK4^WT^ (Fig. 4e). OsMPK4^CA^ was then co-expressed with OsVQ14^WT^ or OsVQ14^T61A^ (where T61 was replaced by A) in tobacco cells. OsVQ14^WT^ or OsVQ14^T61A^ was immunoprecipitated and analyzed by immunoblot with anti-phospho-threonine (anti-pT) antibody. The results showed that OsVQ14^WT^ but OsVQ14^T61A^ was strongly phosphorylated by OsMPK4^CA^ (Fig. 4f). Similarly, when OsVQ32^WT^ or OsVQ32^S141A^ (where S141 was replaced by A) was co-expressed with OsMPK4^CA^, OsVQ32^WT^ was strongly phosphorylated as detected by anti-phospho-serine (anti-pS) antibody, while OsVQ32^S141A^ was only weakly phosphorylated (Fig. 4f). The results suggest that OsMPK4 in vivo phosphorylates OsVQ14 and OsVQ32 mainly on T61 and S141, respectively.

### OsMPK4-Mediated OsVQ14/OsVQ32 Phosphorylation is Required for Rice Defense to *Xoo*

**Fig. 5:**
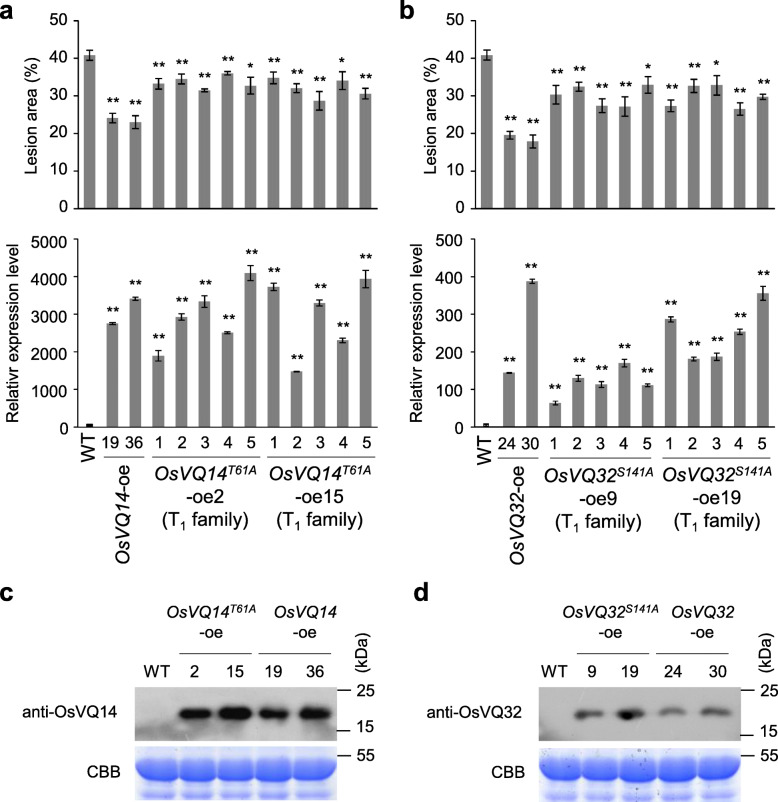
OsVQ14 and OsVQ32 phosphorylations by OsMPK4 contributed to rice resistance to *Xoo*. Bars represent mean (4 to 5 leaves for lesion area and 3 replicates for gene expression) ± SD. The asterisks “**” or “*” indicates a significant difference between transgenic plants and WT plants at *P* < 0.01 or *P* < 0.05. **a** Analyses of lesion area and *OsVQ14* expression level of *OsVQ14*-oe and *OsVQ14*^*T61A*^-oe transgenic plants. **b** Analyses of lesion area and *OsVQ32* expression level of *OsVQ32*-oe and *OsVQ32*^*S141A*^-oe transgenic plants. **c** Analysis of OsVQ14 accumulation of *OsVQ14*-oe and *OsVQ14*^*T61A*^-oe transgenic plants. **d** Analysis of OsVQ32 accumulation of *OsVQ32*-oe and *OsVQ32*^*S141A*^-oe transgenic plants

*OsVQ14*^*T61A*^ and *OsVQ32*^*S141A*^ were overexpressed in WT to determine the effect of OsMPK4-mediated OsVQ14 and OsVQ32 phosphorylation on rice resistance to *Xoo*. Two T_1_ families of each substitution mutant were chosen for resistance evaluation. The defense responses of these transgenic plants were compared with WT and *OsVQ14*-oe or *OsVQ32*-oe following their inoculation with *Xoo*. *OsVQ14*^*T61A*^-oe and *OsVQ32*^*S141A*^-oe plants showed enhanced resistance to *Xoo* compared to the WT, but weakened resistance compared to the non-substituted *OsVQ14*-oe and *OsVQ32*-oe plants (Fig. 5a and b). The protein levels of OsVQ14 and OsVQ32 in those overexpression transgenic lines were also confirmed by immunoblot assays (Fig. 5c and d). These results verified that OsMPK4-mediated OsVQ14 and OsVQ32 phosphorylation is required for rice defense to *Xoo*.

### OsMPKK6 Functions Upstream of the OsMPK4-OsVQ14/OsVQ32 Cascade in Rice Defense to *Xoo*

**Fig. 6:**
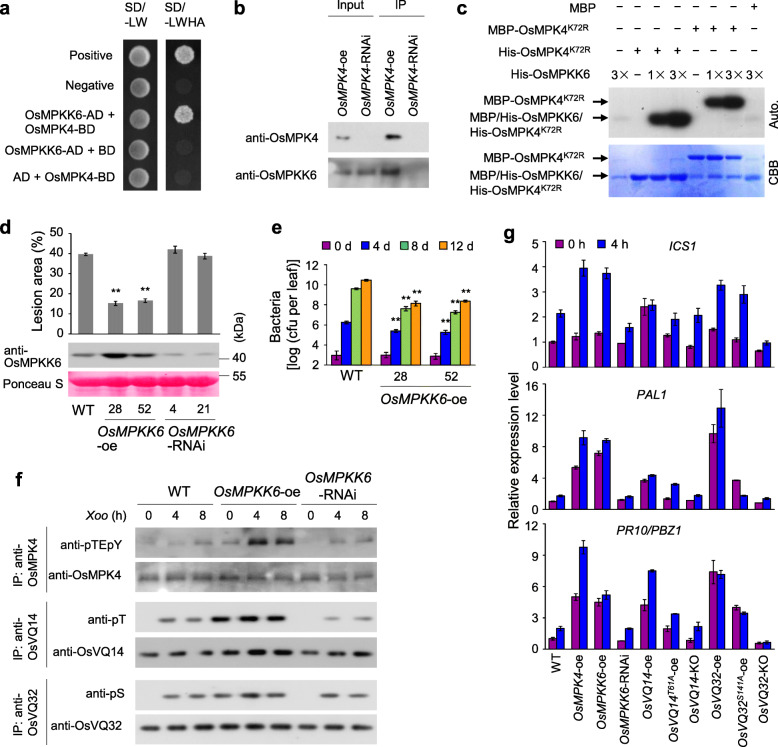
OsMPKK6 promoted OsMPK4-OsVQ14/OsVQ32 cascade phosphorylation in the rice response to *Xoo*. The asterisks “**” indicate a significant difference between transgenic plants and WT plants at *P* < 0.01. **a** Interaction of OsMPK4 with OsMPKK6 in yeast. The interactions were assessed by growing yeast cells on synthetic defined premixes (SD) medium lacking (−) leucine (L), tryptophan (W), histidine (H), and adenine (A). AD, activation domain; BD, DNA-binding domain. Co-transformation of BD-53 and AD-RecT was used as the positive control while co-transformation of BD-Lam and AD-RecT was used as the negative control. **b** Interaction of OsMPK4 with OsMPKK6 in rice plants was analyzed by co-immunoprecipitation assay. Total protein was extracted from rice leaves and anti-OsMPK4 antibody was used for the immunoprecipitation (IP). **c** Phosphorylation assays of OsMPK4^K72R^ by OsMPKK6 in vitro. Auto., Autoradiograph; CBB, Coomassie brilliant blue staining. **d** Analysis of the response of OsMPKK6 transgenic plants to *Xoo* infection. Bars represent mean (5 plants, with each plant having 3 to 5 leaves for lesion area) ± SD. **e** Analysis of the *Xoo* growth in rice leaves. Bars represent mean (3 leaves from 3 positive plants) ± SD. The significant difference was detected between transgenic plants and WT with the same treatment. cfu, colony-forming unit. **f** Analyses of the phosphorylation of OsMPK4, OsVQ14 and OsVQ32 in rice plants before and after *Xoo* infection. 0 h, immediately before *Xoo* inoculation. IP, immunoprecipitated. **g** Analyses of the expression of salicylic acid (SA)-signaling genes before (0 h) and after (4 h) *Xoo* infection. Bars represent the mean (three replicates) ± SD

To identify OsMPK4 interacting proteins, we immunoprecipitated OsMPK4 from *OsMPK4*-oe transgenic plants with anti-OsMPK4 antibody, and then performed liquid chromatography-tandem mass spectrometry (LC-MS/MS) analysis. Five peptides were identified for OsMPKK6 (Fig. S6). It is therefore speculated that OsMPKK6 phosphorylates and activates OsMPK4 in the rice–*Xoo* interaction. Y2H analysis revealed that OsMPKK6 interacted with OsMPK4 in yeast cells (Fig. 6a). Further, OsMPKK6 was detected in immunoprecipitated proteins obtained with anti-OsMPK4 antibody from *OsMPK4*-oe but not *OsMPK4*-RNAi plants (Fig. 6b). In vitro phosphorylation assay showed that His-OsMPKK6 strongly phosphorylated His-OsMPK4^K72R^ or MBP-OsMPK4^K72R^ (the kinase-inactive version of OsMPK4, which was generated by substituting a conserved lysine (K) (K72) residue in the ATP-binding domain for arginine (R)) in a dose-dependent manner (Fig. 6c). These results suggested that OsMPKK6 physically interacts with and phosphorylates OsMPK4.

To determine whether *OsMPKK6* contributes to rice resistance to *Xoo*, we overexpressed and suppressed *OsMPKK6* in WT. The *OsMPKK6*-oe transgenic plants with high accumulation of OsMPKK6 showed increased resistance to *Xoo* compared to that of WT, as indicated by the reduced lesion area and *Xoo* growth (Fig. 6d and e); while the *OsMPKK6-*suppressed transgenic plants (*OsMPKK6*-RNAi) with reduced accumulation of OsMPKK6 showed disease levels similar to that of WT (Fig. 6d), suggesting that OsMPKK6 functions redundantly with other MAPKKs to promote rice resistance to *Xoo*.

To determine whether OsMPKK6 activates OsMPK4, we immunoprecipitated OsMPK4 and assessed its phosphorylation status using the anti-pTEpY antibody, which is widely used to detect MAPKs that have been phosphorylated and activated by MAPKKs (Ma et al. 2017; Willmann et al. 2014). OsMPK4 phosphorylation was increased in WT and *OsMPKK6*-RNAi plants after the inoculation with *Xoo* compared with that in non-inoculated plants (Fig. 6f). However, compared to the WT, OsMPK4 phosphorylation was obviously enhanced in *OsMPKK6*-oe plants before and after *Xoo* infection (Fig. 6f), suggesting that OsMPKK6 promotes OsMPK4 activation in vivo.

We then analyzed OsVQ14 and OsVQ32 phosphorylation in *OsMPKK6* transgenic plants. Immunoblot with anti-pT and anti-pS antibody showed that immunoprecipitated OsVQ14 and OsVQ32 were strongly phosphorylated in *OsMPKK6*-oe transgenic plants compared with that in the WT, both before and after inoculation of *Xoo*. However, OsVQ14 and OsVQ32 phosphorylation in *OsMPKK6*-RNAi plants was similar to that of WT (Fig. 6f). The results suggested that OsMPKK6 promotes OsVQ14 and OsVQ32 phosphorylation in vivo.

Our previous study revealed that overexpression of *OsMPK4* increased the expression of SA-signaling genes (Shen et al. 2010). We wanted to determine whether *OsMPKK6*- and *OsVQ14/32*-mediated rice immunity were also involved in SA signaling pathway. As shown in Fig. 6g, compared to the WT, the transcripts of SA-related genes (*ICS1*, *PAL1*, and *PR10/PBZ1*) were highly increased in *OsMPKK6*- and *OsVQ14/32*-oe plants, normal in *OsMPKK6*-RNAi and *OsVQ14*-KO plants, but reduced in *OsVQ32*-KO plants before and after *Xoo* infection. In addition, the transcripts of these genes were slightly higher in *OsVQ14*^*T61A*^-oe and *OsVQ32*^*S141A*^-oe plants compared with that in WT before and after *Xoo* infection (Fig. 6g). Together, the results suggest that *OsMPKK6*-*OsMPK4*-*OsVQ14/32* form a cascade in SA-involved rice resistance to *Xoo*.

## Discussion

In the present study, we found that OsMPKK6 (the homolog of AtMPKK1, AtMPKK2, and AtMPKK6) interacted with and activated OsMPK4 (the ortholog of AtMPK4) in rice resistance to *Xoo* (Fig. 6a-c, f), resembling the phenomenon observed in *Arabidopsis* whereby AtMPKK1/AtMPKK2 or AtMPKK6 activates AtMPK4 in plant defense signaling (Lian et al. 2018; Qiu et al. 2008). These results implied that the immunity-related MAPK signaling pathway is highly conserved between rice and *Arabidopsis*, providing additional evidence for the conservation of MAPK cascades among eukaryotes (Zhang et al. 2018). The VQ motif-containing proteins OsVQ14 and OsVQ32, the homologs of AtVQ21 (Figs. S4 and S5), functioned as the substrates of the OsMPKK6-OsMPK4 cascade (Figs. 4, 5 and 6) to promote rice resistance to *Xoo* (Figs. 1 and S1). These results partly corroborated the findings of previous studies concerning *Arabidopsis*, that AtMPK4, activated by AtMPKK1/AtMPKK2, phosphorylated AtVQ21 to promote defense response (Andreasson et al. 2005; Qiu et al. 2008). Therefore, our results highlighted a conserved defense-mediated MAPK-VQ cascade between rice and *Arabidopsis*.

As the MAPK-VQ cascade was revealed by the present study to be instrumental in defense signaling transduction, the mechanism by which VQ proteins transmit defense signals emerges as the next topic of focus. Previous results indicated that 29 of the 34 VQ proteins identified in *Arabidopsis* exhibited transcriptional activity in plant cells (Li et al. 2014b), implying that VQ proteins function as transcriptional regulators to transmit the defense signals. However, WRKY transcription factors were more frequently identified as substrates of plant MAPK cascades (Ishihama and Yoshioka 2012; Bigeard and Hirt 2018); for example, WRKY46 functioned as a substrate of the MPK3 to enhance basal plant defense in *Arabidopsis* (Sheikh et al. 2016), OsWRKY45 was identified as the downstream target of OsMPK6 for the positive regulation of rice defense response against *M. oryzae* (Ueno et al. 2015). These results raised some key questions: if both VQ and WRKY are downstream transcription regulators of MAPK cascades, which perform transcriptional reprogramming following signal perception of environmental stresses, and via which mechanisms? Although limited evidence has shown that MPK3/6-targeted VQ proteins interacted with WRKY proteins, thereby affecting the transcriptional activities of the latter to modulate defense gene transcription (Pecher et al. 2014), further research is required to fully understand the underlying mechanisms.

MAPKs phosphorylate their substrates to post-translationally regulate the functions of proteins, thereby contributing to the signaling of multiple environmental stresses and developmental processes (Bigeard and Hirt 2018). Thus, the identification of MAPK substrates will assist significantly in achieving a better understanding of the underlying signaling mechanisms. In the present study, we discovered that two VQ proteins OsVQ14 and OsVQ32 were substrates of OsMPK4 (Figs. 3 and 4), which was highly phosphorylated by OsMPKK6 in vitro and in vivo (Fig. 6c and f). Overexpression of *OsVQ14* or *OsVQ32* enhanced rice resistance to *Xoo* (Fig. 1). Moreover, overexpression of *OsMPKK6* enhanced rice resistance to *Xoo* and simultaneously increased the phosphorylation of OsVQ14 and OsVQ32 (Fig. 6d-f). The results provide not only novel molecular insight into the overall regulatory map of defense signal transduction, but also evidence for breeding new disease-resistant rice varieties via manipulation of these defense-related genes.

## Conclusion

We identified a signaling cascade, OsMPKK6-OsMPK4-OsVQ14/32, that positively regulated rice resistance to *Xoo*. Upon *Xoo* infection, OsMPK4 was phosphorylated and activated by OsMPKK6 to phosphorylate OsVQ14 and OsVQ32 mainly at T61 and S141, respectively, thereby increasing SA-involved rice resistance to *Xoo*.

## Materials and Methods

### Plant Materials

All the transgenic plants in this study are in the genetic background of Zhonghua 11, which belongs to the *japonica/Geng* (*Oryza sativa ssp. japonica/geng*) subgroup of Asian cultivated rice. The *OsMPK4*-RNAi and *OsMPK4*-oe plants (*OsMPK4* was named *OsMPK6* in the original article) have been previously described (Yuan et al. 2007; Shen et al. 2010).

### Rice Transformation

The full-length cDNAs of *OsVQ14*, *OsVQ32*, and *OsMPKK6* were amplified from Zhonghua 11 using the primers listed in Table S2, and inserted into the transformation vector pU1301 (Cao et al. 2007) to construct the overexpressing vector. The cDNA fragment of *OsMPKK6* was amplified using the primers listed in Table S2 and inserted into pDS1301 vector (Yuan et al. 2007) to construct the RNA interference (RNAi) vector of *OsMPKK6*. Two CRISPR gene-targeting units for each *VQ* gene were designed to construct CRISPR gene-editing vectors using the website CRISPR-P (http://cbi.hzau.edu.cn/crispr/) (Liu et al. 2017), then amplified using gene-specific primers (Table S2) and inserted into vector pCXUN-Cas9 (He et al. 2017). All vectors were introduced into the *Agrobacterium tumefaciens* strain EHA105 via electroporation. *Agrobacterium*-mediated transformation was achieved with the calli derived from mature embryos of Zhonghua 11 (Lin and Zhang 2005).

### Pathogen Inoculation

Rice plants were inoculated with 6 *Xoo* strains (one Chinese *Xoo* strain Zhe173 and 5 Philippine *Xoo* strains PXO61, PXO71, PXO99, PXO341, and PXO347) using the leaf-clipping method at the booting stage (Chen et al. 2002) to evaluate the extent of resistance to bacterial blight. The extent of disease was rated by calculating the percentage of the diseased area ((lesion length/leaf length) × 100%) after inoculation. The bacterial growth rate in the rice leaves was measured by counting the colony-forming units (Sun et al. 2004).

### Gene Expression

Rice flag leaves were sampled at the booting stage, and immediately frozen in liquid nitrogen prior to storage at − 80 °C for ribonucleic acid (RNA) isolation. Reverse transcription quantitative PCR (RT-qPCR) was conducted as previously described (Qiu et al. 2007). The primers used for RT-qPCR are listed in Table S2. The expression level of the rice *actin* gene was used as an internal control to normalize the expression value for each gene.

### Protein–Protein Interaction

The Y2H assays were conducted with Matchmaker GAL4 Two-Hybrid System according to the manufacturer’s manual (Clontech, Winsconsin, USA). Briefly, different cDNA fragments of *OsVQ14*, *OsVQ32*, and their truncations were amplified from rice variety Zhonghua 11 with specific primers (Table S2) and cloned into vector pGADT7 with the GAL4 activation domain. N-terminal deletion 1 (OsVQ14(32)-dN1) lacks domain I, which contains a putative MAPK docking domain (Andreasson et al. 2005). N-terminal deletion 2 (OsVQ14(32)-dN2) lacks both domain I and a VQ domain. C-terminal deletion 1 (OsVQ14(32)-dC1) contains inter N-terminal and domain I. C-terminal deletion 2 (OsVQ14(32)-dC2) only lacks the most C-terminal amino acids until the VQ domain. OsVQ14(32)-dNC lacks both N- and C-terminals, but remains the VQ domain of both VQ proteins. The cDNAs of *OsMPK4* and *OsMPKK6* were amplified from Zhonghua 11 and cloned into vector pGBKT7 (with the GAL4 DNA-binding domain) and pGADT7, respectively. The interactions between proteins were assessed based on the growth of yeast cells on a synthetic defined premixes (SD) medium lacking (−) leucine (L), tryptophan (W), histidine (H), and adenine (A).

Co-immunoprecipitation assays were conducted following a previously reported procedure (Ma et al. 2017) to investigate proteins interaction in rice plants. Total proteins were extracted from rice leaves with extraction buffer (50 mM Tris-HCl (pH 7.5), 5 mM EDTA (pH 8.0), 5 mM EGTA (pH 7.0), 5 mM Na_3_VO_4_, 10 mM NaF, 50 mM β-glycerophosphate, 10% glycerol, 1 mM PMSF, and complete EDTA Free protease inhibitor cocktail) (Roche, China), and precleared with protein A/G agarose mixture (Roche, China) for 2 h at 4 °C. The cleaned proteins were then transferred and incubated with antibody overnight at 4 °C, and added protein A/G agarose mixture for further incubation for 3 h at 4 °C. The immunocomplex was washed three times with wash buffer (50 mM Tris-HCl (pH 7.5), 150 mM NaCl, 5 mM EDTA, 10 mM NaF, 5 mM Na_3_VO_4_, 50 mM β-glycerophosphate, 0.1% Tween 20, 1 mM PMSF, and complete EDTA-free protease inhibitor cocktail). Finally, the samples were boiled with 5× SDS-PAGE loading buffer for 5 min and subjected to immunoblot analysis.

### Protein Point Mutation

PCR-mediated site mutagenesis was performed with site-directed Mutagenesis Kit (Sangon, China) to introduce point mutations into the open reading frames (ORFs) of *OsVQ14*, *OsVQ32*, and *OsMPK4* using the primers listed in Table S2, in accordance with the manufacturer’s protocol.

### Protein Expression, Purification and Phosphorylation Assays in Vitro

To express recombinant proteins in *E. coli*, the ORFs of *OsMPKK6*, *OsMPK4*, and *OsMPK4*^*CA*^ were cloned into the pET28 vector (Invitrogen, USA), the ORF of *OsMPK4*^*K72R*^ was cloned into the vectors pET28 and pMAL-c2x (New England Biolabs, USA), and the ORFs of Os*VQ14*, Os*VQ32*, and their mutation versions were cloned into the pCOLD-TF vector (Takara, China). The *E. coli* cells were cultivated in Lysogeny Broth containing 100 μg/mL ampicillin or 50 μg/mL kanamycin at 37 °C and shaken for 2 to 3 h until the required optical density was reached (OD_600_ to 0.6–1.0). Expression was induced by adding 0.1% Isopropyl β-D-1-thiogalactopyranoside for 16–18 h at 16 °C. Proteins were purified for a direct phosphorylation in vitro in the presence of γ-^32^P-ATP, as previously described (Ma et al. 2017).

### Immunoprecipitated Protein Phosphorylation Assay

To express proteins in tobacco (*Nicotiana benthamiana*) cells, plasmids were transformed into tobacco plants via *A. tumefaciens* strain GV3101-pM90. The immunoprecipitated protein phosphorylation assay was performed as described previously (Ma et al. 2017). Briefly, total proteins were extracted from tobacco or rice leaves with extraction buffer as described above. Target protein was immunoprecipitated using its specific antibody and protein A/G agarose. The immunocomplex was washed twice with wash buffer 1 (extraction buffer containing 150 mM NaCl, 0.1% Tween 20), twice with wash buffer 2 (extraction buffer containing 500 mM NaCl, 0.1% Tween 20), and once with wash buffer 3 (extraction buffer containing 0.1% Tween 20). Western blot analysis was then performed to detect protein phosphorylation and protein levels using phospho-specific antibodies (anti-pTEpY antibody and anti-pT antibody, Cell Signaling Technology; anti-pS antibody, Abcam) and non-phospho antibodies (anti-OsMPKK6 and anti-OsMPK4 antibodies that were produced using 6× His-tagged OsMPKK6 and OsMPK4 as antigens, respectively; anti-OsVQ14 and anti-OsVQ32 antibodies that were produced using TF- and His-tagged OsVQ14 and OsVQ32 as antigens, respectively), respectively.

### Phylogenetic Tree Construction

The proteins used for phylogenetic tree construction were listed in Tables S3 and S4 (Cheng et al. 2012; Li et al. 2014a). Unrooted tree was constructed using MEGA-X with neighbor-joining method based on Dayhoff model (Kumar et al. 2018). The gaps or missing data were treated as partial deletion with coverage cutoff at 50%. Bootstrap method with 1000 bootstrap replications was used to test the phylogeny. An online tool iTOL (Interactive Tree of Life, https://itol.embl.de/) was used to annotate the tree (Letunic and Bork 2019).

### Statistical Analysis

The significance of the differences between the control and treatment was analyzed using the pair-wise *t*-test function installed in the Microsoft Office Excel program. The correlations between the disease and gene expression level was analyzed using the Pearson correlation method with GraphPad Prism 5 software.

## Additional Files

**Additional file 1: Fig. S1.** Overexpressing *OsVQ14* and *OsVQ32* enhanced rice resistance to *Xoo*. Bars represent mean (3 to 5 leaves of lesion area for each plant, and 3 replicates for expression level) ± standard deviation (SD). The asterisks “**” or “*” indicate a significant difference between transgenic plants and wild type (WT; Zhonghua 11) plants at *P* < 0.01 or *P* < 0.05, respectively. N: negative transgenic plants. **Fig. S2.** The positions of CRISPR/Cas9 system target sites in two VQ genes and sequencing results of transgenic plants. The protospacer adjacent motif (PAM) (CCN) is shown in bold and underlined. The dashed lines indicate base pairs deletion. Zhonghua 11 (WT) is the background of transgenic plants. Rectangles “I” and “VQ” represent domain I and VQ domain, respectively. **a** The two CRISPR/Cas9 system target sites (TS) in *OsVQ14*. **b** Sequencing results of *OsVQ14*-KO plants. “ … (51bp) … ” means there are 51 base pairs and “ … (17 aa) … ” means there are 17 amino acids. **c** The two CRISPR/Cas9 system target sites (TS) in *OsVQ32*. **d** Sequencing results of *OsVQ32*-KO plants, “ … (30bp) … ” means there are 30 base pairs and “ … (10 aa) … ” means there are 10 amino acids. **Fig. S3.** The sequencing results of off-target sites of target site 1 **(a)** and 2 **(b)** in *OsVQ32*-KO88, *OsVQ32*-KO91, and the WT. The protospacer adjacent motif (PAM) (CCN) are in bold and underlined. The putative off-target sites are indicated with rectangles. **Fig. S4.** Phylogenetic tree of VQ proteins in *Arabidopsis* and rice. This phylogenetic unrooted tree was constructed using MEGA-X with neighbor-joining (NJ) method based on Dayhoff model. The gaps or missing data treatment was set as Partial deletion with coverage cutoff at 50%. Bootstrap method with 1000 bootstrap replications was used to test the phylogeny. An online tool iTOL (Interactive Tree of Life, https://itol.embl.de/) was used to annotate the tree. Only those values greater than 40% are displayed. The transcripts ID encoding VQ proteins in rice and *Arabidopsis* are listed in Table S3 and Table S4, respectively. **Fig. S5.** Sequence alignment of AtVQ21, OsVQ14 and OsVQ32. The abbreviations, dC1, dC2, dNC, dN1, dN2, on the top left and top right of triangles represent the start and end amino acids of each truncated proteins, respectively. Domains I is shown by underline. Amino acids deletions in mutant plants are labeled by dotted lines. Amino acids identical in two proteins are blue, amino acids identical in three proteins are shaded, and residues similar in two proteins are pink. The putative nuclear localization sequences are red. Putative MAP kinase phosphorylation sites (S/TP) are underlined. Asterisks indicate the highly conserved FxxxVQxLTG (x, any amino acid) sequences. The amino acids of truncated OsVQ14 and OsVQ32 are indicated by black and red colored triangles, respectively, which are corresponding to the truncated OsVQ14 and OsVQ32 in Fig. 3a and b. **Fig. S6.** Amino acid sequences of OsMPKK6. The boxed text indicates peptides found by mass spectrometry.

**Additional file 2 Table S1.** The putative off-target sites of CRISPR/Cas9 system in *OsVQ32*-KO plants. **Table S2.** Primers used in vector construction, gene expression analysis, and detection of positive transgenic plants. **Table S3.** Rice *VQ* genes used in phylogenetic tree. **Table S4.** *Arabidopsis VQ* genes used in phylogenetic tree.

## Data Availability

All data generated or analyzed during this study are included in this published article and its supplementary information files.

## Abbreviations

VQ: Valine-glutamine
MAPK: Mitogen-activated protein kinase
MAPKK: Mitogen-activated protein kinase kinase
MAPKKK: Mitogen-activated protein kinase kinase kinase
*Xoo*: *Xanthomonas oryzae* pv. *oryzae*
MR: Major disease resistance
QTL: Quantitative trait loci
CRISPR/Cas9: Clustered Regularly Interspaced Short Palindromic Repeats/CRISPR-associated protein 9
Y2H: Yeast two-hybrid
LC-MS/MS: Liquid chromatography-tandem mass spectrometry
RT-qPCR: Reverse transcription quantitative PCR
ORF: Open reading frames
