# Supplementary material for: Two VQ Proteins are Substrates of the OsMPKK6-OsMPK4 Cascade in Rice Defense Against Bacterial Blight
Source: Rice (N Y). 2021 Apr 28;14:39. doi: 10.1186/s12284-021-00483-y (PMC8081811; doi:10.1186/s12284-021-00483-y)
Supplement: Supplementary file 1 — Additional file 1: Fig. S1. Overexpressing OsVQ14 and OsVQ32 enhanced rice resistance to Xoo. Bars represent mean (3 to 5 leaves of lesion area for each plant, and 3 replicates for expression level) ± standard deviation (SD). The asterisks “**” or “*” indicate a significant difference between transgenic plants and wild type (WT; Zhonghua 11) plants at P < 0.01 or P < 0.05, respectively. N: negative transgenic plants. Fig. S2. The positions of CRISPR/Cas9 system target sites in two VQ genes and sequencing results of transgenic plants. The protospacer adjacent motif (PAM) (CCN) is shown in bold and underlined. The dashed lines indicate base pairs deletion. Zhonghua 11 (WT) is the background of transgenic plants. Rectangles “I” and “VQ” represent domain I and VQ domain, respectively. a The two CRISPR/Cas9 system target sites (TS) in OsVQ14. b Sequencing results of OsVQ14-KO plants. “ … (51bp) … ” means there are 51 base pairs and “ … (17 aa) … ” means there are 17 amino acids. c The two CRISPR/Cas9 system target sites (TS) in OsVQ32. d Sequencing results of OsVQ32-KO plants, “ … (30bp) … ” means there are 30 base pairs and “ … (10 aa) … ” means there are 10 amino acids. Fig. S3. The sequencing results of off-target sites of target site 1 (a) and 2 (b) in OsVQ32-KO88, OsVQ32-KO91, and the WT. The protospacer adjacent motif (PAM) (CCN) are in bold and underlined. The putative off-target sites are indicated with rectangles. Fig. S4. Phylogenetic tree of VQ proteins in Arabidopsis and rice. This phylogenetic unrooted tree was constructed using MEGA-X with neighbor-joining (NJ) method based on Dayhoff model. The gaps or missing data treatment was set as Partial deletion with coverage cutoff at 50%. Bootstrap method with 1000 bootstrap replications was used to test the phylogeny. An online tool iTOL (Interactive Tree of Life, https://itol.embl.de/) was used to annotate the tree. Only those values greater than 40% are displayed. The transcripts ID encoding VQ proteins in rice [file 12284_2021_483_MOESM1_ESM.pptx]

## Slide 1
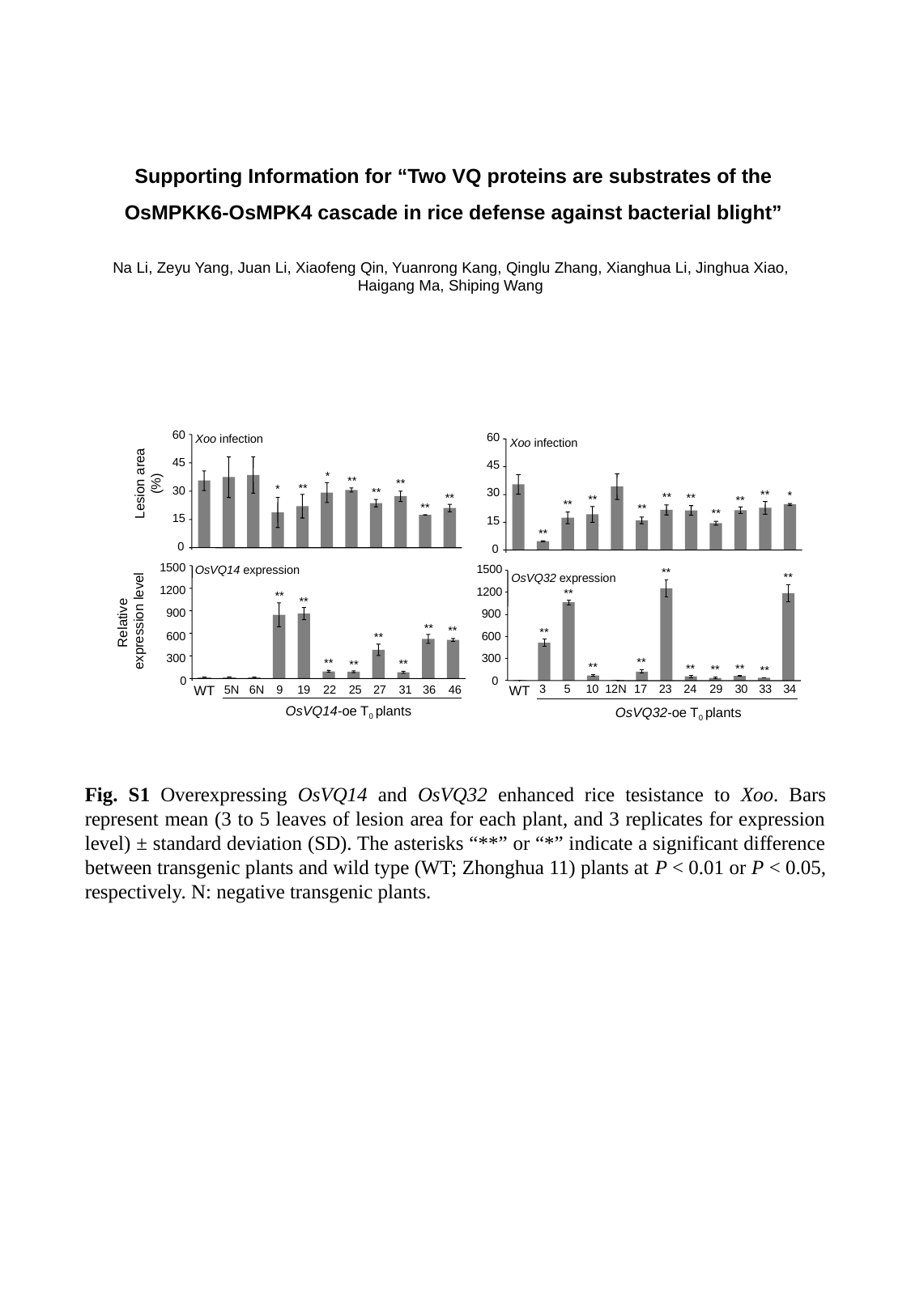

Supporting Information for “Two VQ proteins are substrates of the OsMPKK6-OsMPK4 cascade in rice defense against bacterial blight”
Na Li, Zeyu Yang, Juan Li, Xiaofeng Qin, Yuanrong Kang, Qinglu Zhang, Xianghua Li, Jinghua Xiao, Haigang Ma, Shiping Wang
Xoo infection
60
Lesion area (%)
45
*
**
**
**
**
**
**
*
30
15
0
OsVQ14 expression
Relative
 expression level
1500
1200
**
**
**
**
**
**
**
**
900
600
300
0
WT
5N
6N
9
19
22
25
27
31
36
46
OsVQ14-oe T0 plants
60
Xoo infection
45
30
**
*
**
**
**
**
**
**
**
**
15
0
1500
OsVQ32 expression
**
**
**
**
**
**
**
**
**
**
1200
900
600
300
0
3
5
10
12N
17
23
24
29
30
33
34
WT
OsVQ32-oe T0 plants
Fig. S1 Overexpressing OsVQ14 and OsVQ32 enhanced rice tesistance to Xoo. Bars represent mean (3 to 5 leaves of lesion area for each plant, and 3 replicates for expression level) ± standard deviation (SD). The asterisks “**” or “*” indicate a significant difference between transgenic plants and wild type (WT; Zhonghua 11) plants at P < 0.01 or P < 0.05, respectively. N: negative transgenic plants.

## Slide 2
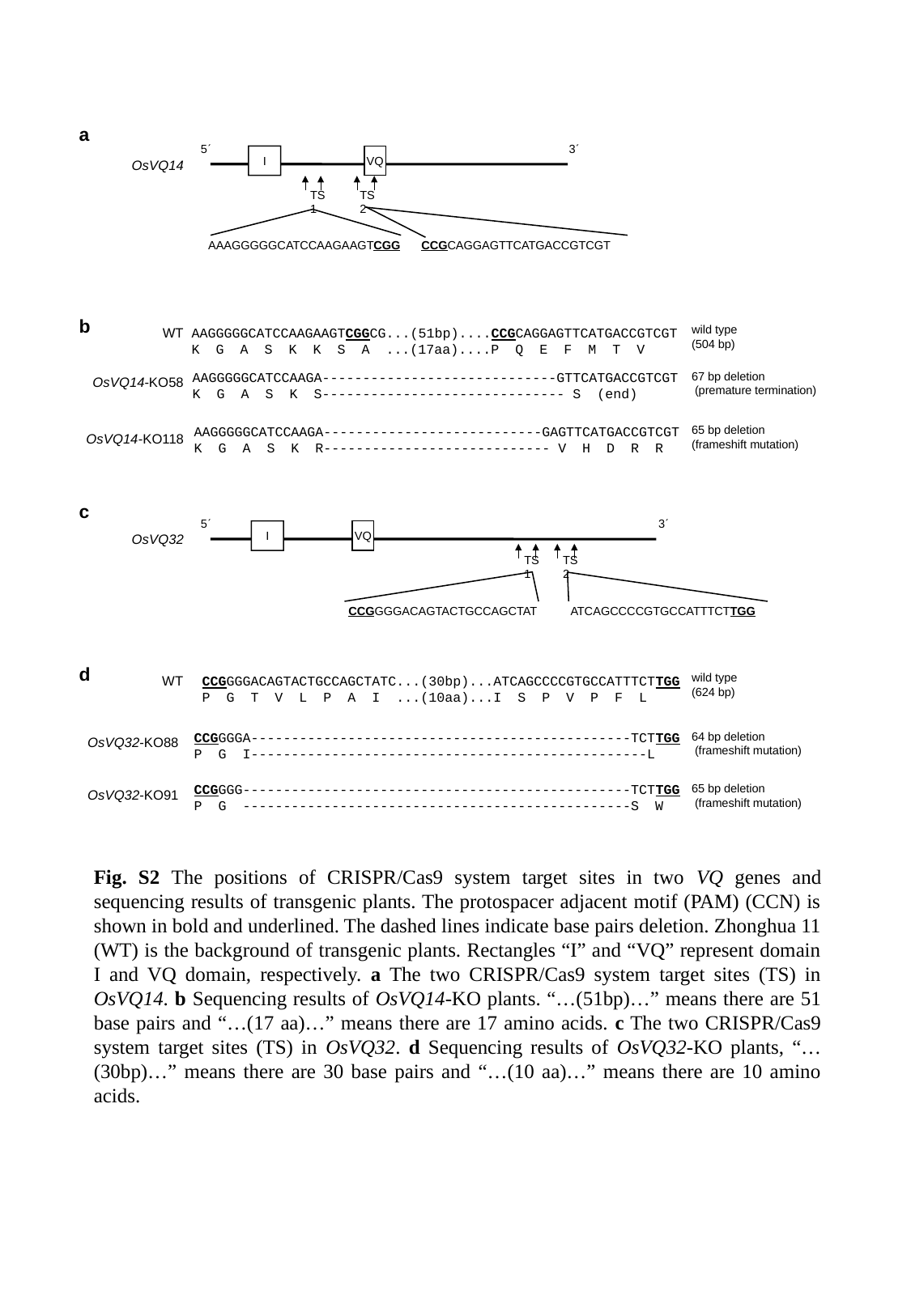

a
5ˊ
3ˊ
I
VQ
OsVQ14
TS2
TS1
AAAGGGGGCATCCAAGAAGTCGG
CCGCAGGAGTTCATGACCGTCGT
b
wild type (504 bp)
WT
 AAGGGGGCATCCAAGAAGTCGGCG...(51bp)....CCGCAGGAGTTCATGACCGTCGT
 K G A S K K S A ...(17aa)....P Q E F M T V
AAGGGGGCATCCAAGA-----------------------------GTTCATGACCGTCGT
K G A S K S------------------------------ S (end)
67 bp deletion
 (premature termination)
OsVQ14-KO58
65 bp deletion
(frameshift mutation)
AAGGGGGCATCCAAGA---------------------------GAGTTCATGACCGTCGT
K G A S K R---------------------------- V H D R R
OsVQ14-KO118
c
3ˊ
5ˊ
I
VQ
OsVQ32
TS2
TS1
CCGGGGACAGTACTGCCAGCTAT
ATCAGCCCCGTGCCATTTCTTGG
d
wild type (624 bp)
WT
 CCGGGGACAGTACTGCCAGCTATC...(30bp)...ATCAGCCCCGTGCCATTTCTTGG
 P G T V L P A I ...(10aa)...I S P V P F L
CCGGGGA-----------------------------------------------TCTTGG
P G I-------------------------------------------------L
64 bp deletion
 (frameshift mutation)
OsVQ32-KO88
CCGGGG------------------------------------------------TCTTGG
P G ------------------------------------------------S W
65 bp deletion
 (frameshift mutation)
OsVQ32-KO91
Fig. S2 The positions of CRISPR/Cas9 system target sites in two VQ genes and sequencing results of transgenic plants. The protospacer adjacent motif (PAM) (CCN) is shown in bold and underlined. The dashed lines indicate base pairs deletion. Zhonghua 11 (WT) is the background of transgenic plants. Rectangles “I” and “VQ” represent domain I and VQ domain, respectively. a The two CRISPR/Cas9 system target sites (TS) in OsVQ14. b Sequencing results of OsVQ14-KO plants. “…(51bp)…” means there are 51 base pairs and “…(17 aa)…” means there are 17 amino acids. c The two CRISPR/Cas9 system target sites (TS) in OsVQ32. d Sequencing results of OsVQ32-KO plants, “…(30bp)…” means there are 30 base pairs and “…(10 aa)…” means there are 10 amino acids.

## Slide 3
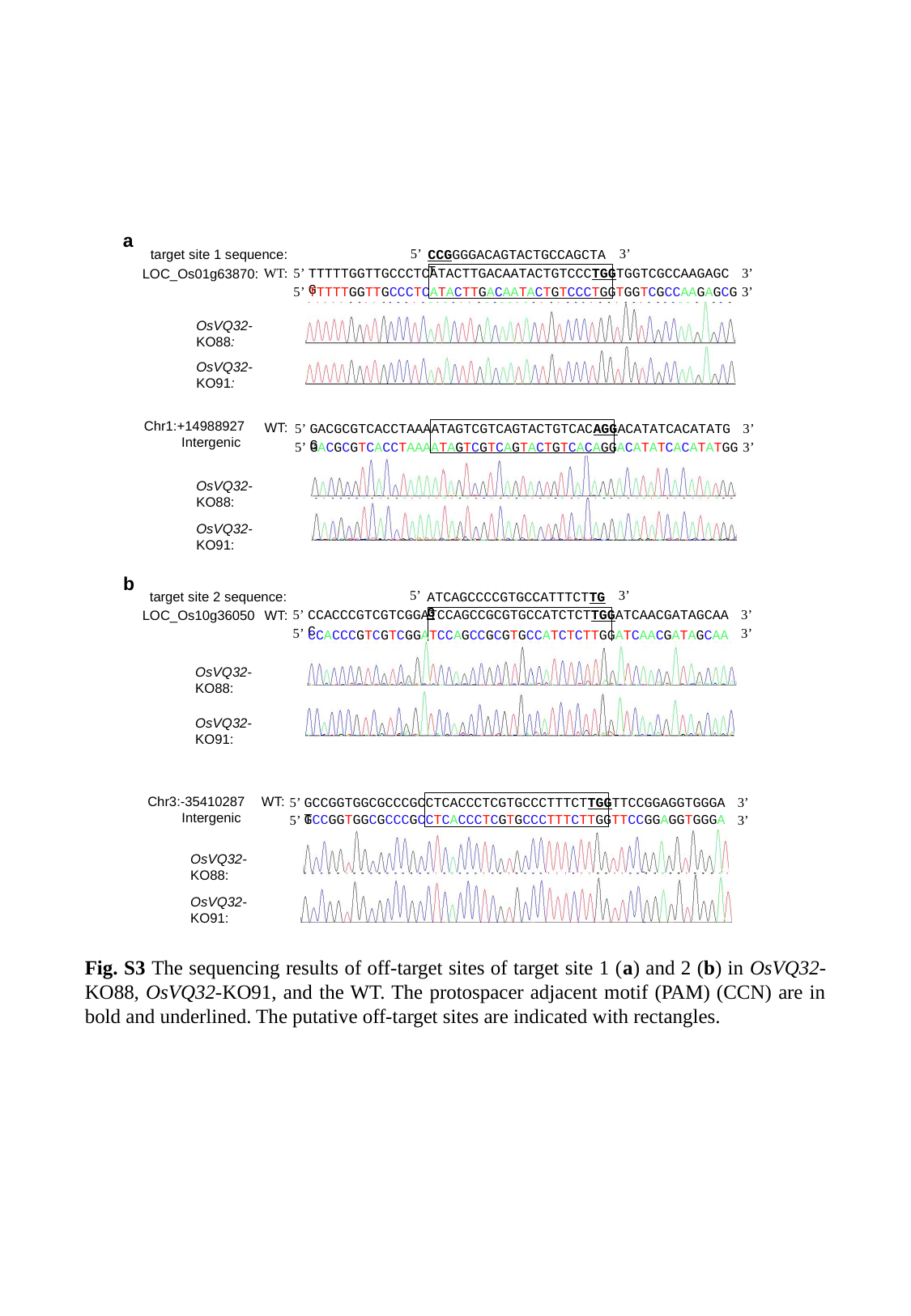

a
5’
3’
target site 1 sequence:
CCGGGGACAGTACTGCCAGCTAT
5’
tttttggttgccctcatacttgacaatactgtccctggtggtcgccaagagcg
3’
WT:
LOC_Os01g63870:
5’
tttttggttgccctcatacttgacaatactgtccctggtggtcgccaagagcg
3’
OsVQ32-KO88:
OsVQ32-KO91:
Chr1:+14988927
 Intergenic
WT:
5’
GACGCGTCACCTAAAATAGTCGTCAGTACTGTCACAGGACATATCACATATGG
3’
5’
GACGCGTCACCTAAAATAGTCGTCAGTACTGTCACAGGACATATCACATATGG
3’
OsVQ32-KO88:
OsVQ32-KO91:
b
5’
3’
target site 2 sequence:
ATCAGCCCCGTGCCATTTCTTGG
5’
CCACCCGTCGTCGGATCCAGCCGCGTGCCATCTCTTGGATCAACGATAGCAAC
3’
LOC_Os10g36050
WT:
5’
3’
CCACCCGTCGTCGGATCCAGCCGCGTGCCATCTCTTGGATCAACGATAGCAAC
OsVQ32-KO88:
OsVQ32-KO91:
Chr3:-35410287
 Intergenic
WT:
5’
GCCGGTGGCGCCCGCCTCACCCTCGTGCCCTTTCTTGGTTCCGGAGGTGGGAT
3’
GCCGGTGGCGCCCGCCTCACCCTCGTGCCCTTTCTTGGTTCCGGAGGTGGGAT
5’
3’
OsVQ32-KO88:
OsVQ32-KO91:
Fig. S3 The sequencing results of off-target sites of target site 1 (a) and 2 (b) in OsVQ32-KO88, OsVQ32-KO91, and the WT. The protospacer adjacent motif (PAM) (CCN) are in bold and underlined. The putative off-target sites are indicated with rectangles.

## Slide 4
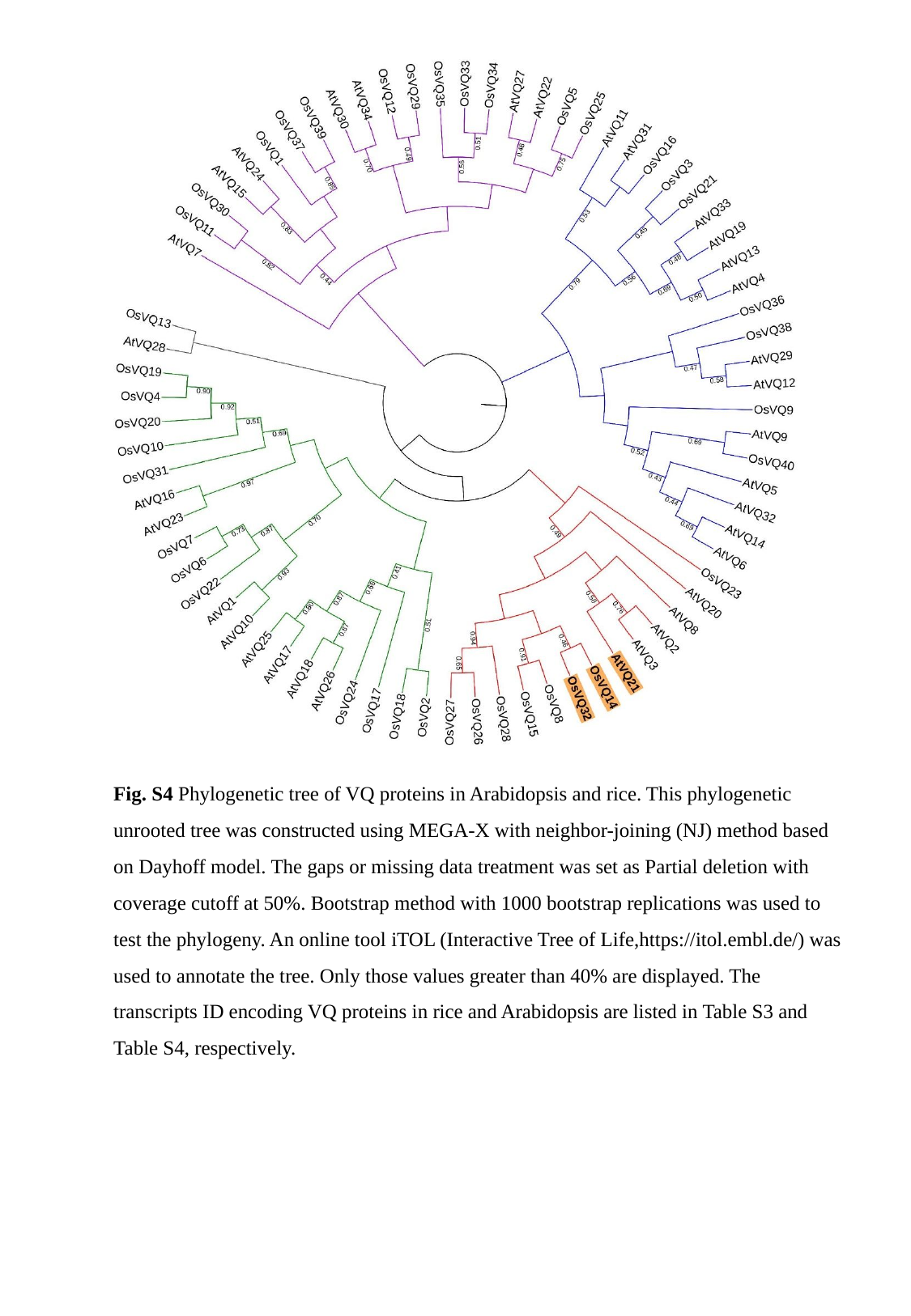

Fig. S4 Phylogenetic tree of VQ proteins in Arabidopsis and rice. This phylogenetic unrooted tree was constructed using MEGA-X with neighbor-joining (NJ) method based on Dayhoff model. The gaps or missing data treatment was set as Partial deletion with coverage cutoff at 50%. Bootstrap method with 1000 bootstrap replications was used to test the phylogeny. An online tool iTOL (Interactive Tree of Life,https://itol.embl.de/) was used to annotate the tree. Only those values greater than 40% are displayed. The transcripts ID encoding VQ proteins in rice and Arabidopsis are listed in Table S3 and Table S4, respectively.

## Slide 5
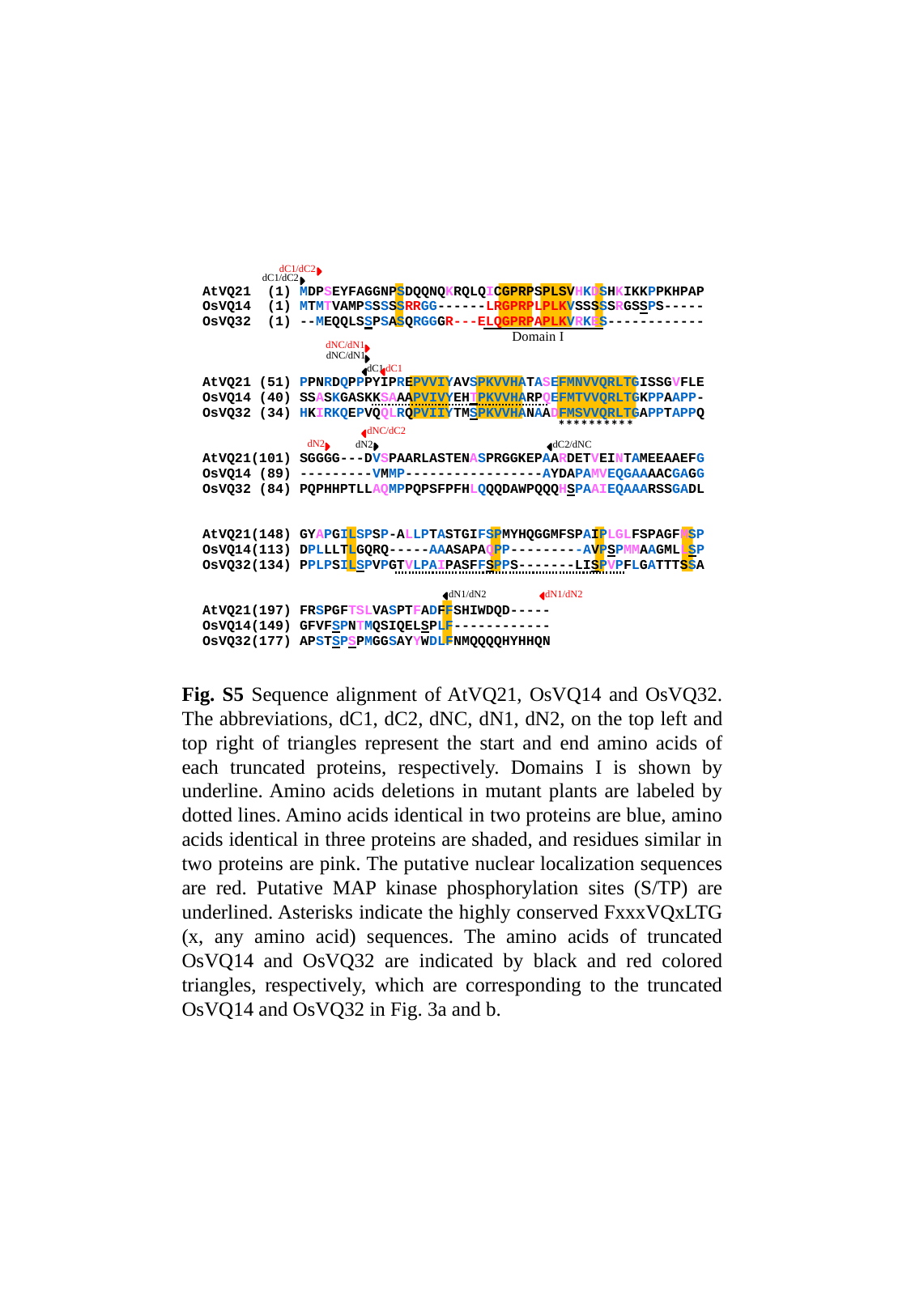

dC1/dC2
dC1/dC2
 AtVQ21 (1) MDPSEYFAGGNPSDQQNQKRQLQICGPRPSPLSVHKDSHKIKKPPKHPAP
 OsVQ14 (1) MTMTVAMPSSSSSRRGG------LRGPRPLPLKVSSSSSRGSSPS-----
 OsVQ32 (1) --MEQQLSSPSASQRGGGR---ELQGPRPAPLKVRKES------------
 AtVQ21 (51) PPNRDQPPPYIPREPVVIYAVSPKVVHATASEFMNVVQRLTGISSGVFLE
 OsVQ14 (40) SSASKGASKKSAAAPVIVYEHTPKVVHARPQEFMTVVQRLTGKPPAAPP-
 OsVQ32 (34) HKIRKQEPVQQLRQPVIIYTMSPKVVHANAADFMSVVQRLTGAPPTAPPQ
 AtVQ21(101) SGGGG---DVSPAARLASTENASPRGGKEPAARDETVEINTAMEEAAEFG
 OsVQ14 (89) ---------VMMP-----------------AYDAPAMVEQGAAAACGAGG
 OsVQ32 (84) PQPHHPTLLAQMPPQPSFPFHLQQQDAWPQQQHSPAAIEQAAARSSGADL
 AtVQ21(148) GYAPGILSPSP-ALLPTASTGIFSPMYHQGGMFSPAIPLGLFSPAGFMSP
 OsVQ14(113) DPLLLTLGQRQ-----AAASAPAQPP---------AVPSPMMAAGMLLSP
 OsVQ32(134) PPLPSILSPVPGTVLPAIPASFFSPPS-------LISPVPFLGATTTSSA
 AtVQ21(197) FRSPGFTSLVASPTFADFFSHIWDQD-----
 OsVQ14(149) GFVFSPNTMQSIQELSPLF------------
 OsVQ32(177) APSTSPSPMGGSAYYWDLFNMQQQQHYHHQN
Domain I
dNC/dN1
dNC/dN1
dC1
dC1
*
*
*
*
*
*
*
*
*
*
dNC/dC2
dN2
dN2
dC2/dNC
dN1/dN2
dN1/dN2
Fig. S5 Sequence alignment of AtVQ21, OsVQ14 and OsVQ32. The abbreviations, dC1, dC2, dNC, dN1, dN2, on the top left and top right of triangles represent the start and end amino acids of each truncated proteins, respectively. Domains I is shown by underline. Amino acids deletions in mutant plants are labeled by dotted lines. Amino acids identical in two proteins are blue, amino acids identical in three proteins are shaded, and residues similar in two proteins are pink. The putative nuclear localization sequences are red. Putative MAP kinase phosphorylation sites (S/TP) are underlined. Asterisks indicate the highly conserved FxxxVQxLTG (x, any amino acid) sequences. The amino acids of truncated OsVQ14 and OsVQ32 are indicated by black and red colored triangles, respectively, which are corresponding to the truncated OsVQ14 and OsVQ32 in Fig. 3a and b.

## Slide 6
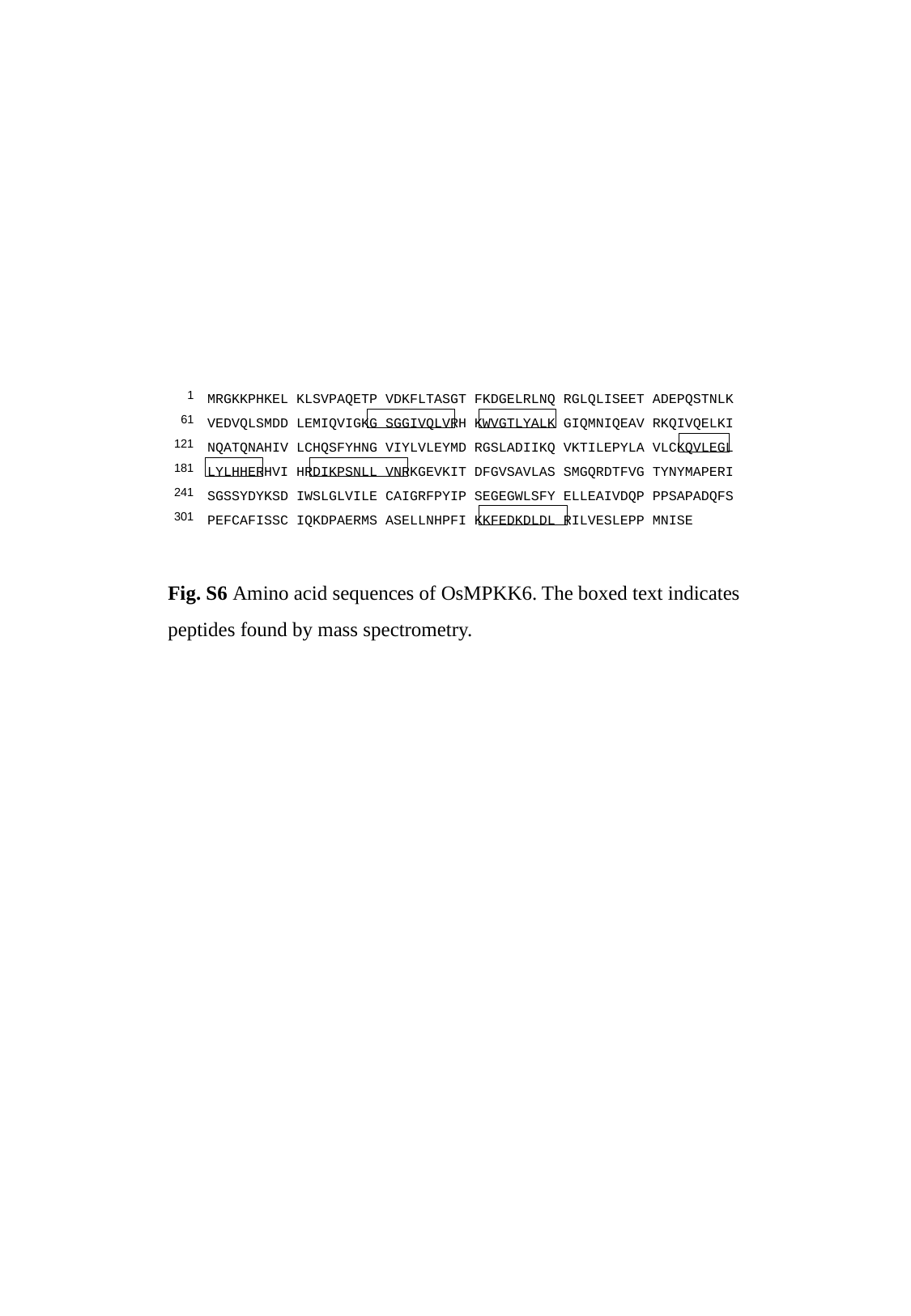

MRGKKPHKEL KLSVPAQETP VDKFLTASGT FKDGELRLNQ RGLQLISEET ADEPQSTNLK
VEDVQLSMDD LEMIQVIGKG SGGIVQLVRH KWVGTLYALK GIQMNIQEAV RKQIVQELKI
NQATQNAHIV LCHQSFYHNG VIYLVLEYMD RGSLADIIKQ VKTILEPYLA VLCKQVLEGL
LYLHHERHVI HRDIKPSNLL VNRKGEVKIT DFGVSAVLAS SMGQRDTFVG TYNYMAPERI
SGSSYDYKSD IWSLGLVILE CAIGRFPYIP SEGEGWLSFY ELLEAIVDQP PPSAPADQFS
PEFCAFISSC IQKDPAERMS ASELLNHPFI KKFEDKDLDL RILVESLEPP MNISE
1
61
121
181
241
301
Fig. S6 Amino acid sequences of OsMPKK6. The boxed text indicates peptides found by mass spectrometry.
