# Supplementary material for: Two VQ Proteins are Substrates of the OsMPKK6-OsMPK4 Cascade in Rice Defense Against Bacterial Blight
Source: Rice (N Y). 2021 Apr 28;14:39. doi: 10.1186/s12284-021-00483-y (PMC8081811; doi:10.1186/s12284-021-00483-y)
Supplement: Supplementary file 2 — Additional file 2 Table S1. The putative off-target sites of CRISPR/Cas9 system in OsVQ32-KO plants. Table S2. Primers used in vector construction, gene expression analysis, and detection of positive transgenic plants. Table S3. Rice VQ genes used in phylogenetic tree. Table S4. Arabidopsis VQ genes used in phylogenetic tree. [file 12284_2021_483_MOESM2_ESM.docx]

**Table S1.** The putative off-target sites of CRISPR/Cas9 system in *OsVQ32*-KO plants.

| **Target name** | **Target sequence(5'-3')** | **Putative off-target site (5'-3')** | **Locus** | **Putative off-target gene/region** |
| --- | --- | --- | --- | --- |
| Unit I off-target 1 | CCGGGGACAGTACTGCCAGCTAT | ATACTTGACAATACTGTCCCTGG | Chr1:+37064121 | LOC_Os01g63870 |
| Unit I off-target 2 |  | ATAGTCGTCAGTACTGTCACAGG | Chr1:+14988927 | Intergenic |
| Unit II off-target 1 | ATCAGCCCCGTGCCATTTCTTGG | TCCAGCCGCGTGCCATCTCTTGG | Chr10:+19260891 | LOC_Os10g36050 |
| Unit II off-target 2 |  | CTCACCCTCGTGCCCTTTCTTGG | Chr3:-35410287 | Intergenic |

**Table S2.** Primers used in vector construction, gene expression analysis, and detection of positive transgenic plants.

| Gene name  (RGAP code)^a^ | Primer name | Forward primer (5’-3’) | Reverse primer(5’-3’) | Use |
| --- | --- | --- | --- | --- |
| *OsMPKK6*  (Os01g32660)  *OsMPK4*  (Os01g32660)  *OsVQ14*  (Os03g57520)  *OsVQ32*  (Os08g01260)  *ICS1*  (Os09g19734)  *PAL1*  (Os02g41630)  *PR10/PBZ1*  (Os12g36880)  *Actin*  (Os03g50885) | OMKK6-F1/R1  dsKK6F/R  OMKK6-28a-F/R  MPK6Y2HF1/R1  MPK6-K72R-F1/R1  MPK6-CA-F/R  OsMKS6F1/Y2HR1  OsMKS6C1F/R  OsMKS6C2F/R  OsMKS6Y2HF1/R1  OsMKS6Y2HF1/JDR1  OsMKS6Y2HF1/JDR2  OsMKS6JDF1/R2  OsMKS6JDF1/Y2HR1  OsMKS6JDF2/Y2HR1  OsMKS6F1/mtR5  OsMKS6mtF1/R1  OsMKS6mtF2/R2  OsMKS6mtF4/R4  MKS6-rtMHG-F/R  OsMKS5F1/Y2HR1  OsMKS5C1F/R  OsMKS5C2F/R  OsMKS5Y2HF1/R1  OsMKS5Y2HF1/JDR1  OsMKS5Y2HF1/JDR2  OsMKS5JDF1/R2  OsMKS5JDF1/Y2HR1  OsMKS5JDF2/Y2HR1  OsMKS5mtF1/Y2HR1  OsMKS5mtF2/R2  OsMKS5mtF3/R3  OsMKS5mtF4/R4  OsMKS5mtF5/R5  OsMKS5mtF6/R6  MKS5-rtMHG-F/R  ICS1-Realtime-F/R  PAL1-Realtime-F/R  PR10-Realtime-F/R  actin120F/R | GGTACCATGAGGGGGAAGAAGCCGC^b^  AGACTAGTGGTACCGCCACCAATCTTTCTACCA^c^  AAGAATTCATGAGGGGGAAGAAGCCGC^e^  TCGAATTCATGGATTCCTCCTCCGGCG^e^  GGAAGTTGCCATCCGGAAGA^g^  AGACTGGCCTCATGACAGCGTATG^g^  TCGGTACCATGACCATGACAGTGGCCAT^b^  ACGACGGTCATGAACTCCTGGTTTTAGAGCTAGAAATAGCAAGTTA  AAAGGGGGCATCCAAGAAGTGTTTTAGAGCTAGAAATAGCAAGTTA  TCGAATTCATGACCATGACAGTGGCCAT^e^  the same as above  the same as above  CCGGAATTCAAGAAGTCGGCGGCGGC^e^  CCGGAATTCAAGAAGTCGGCGGCGGC^e^  CCGGAATTCGTGATGATGCCGGCGTACG^e^  the same as above  GAGGGGGTCGGCGCCGTCGTCGTCGGCGTC  GTACGAGCACGCGCCCAAGGTGGTGCACGC  CATGCTGCTGGCGCCGGGCTTCGTCTTCGCCCCGAACACCATGCAGTC  GTCGTCGGCGTCAAAGG  CGGGGTACCATGGAGCAGCAGTTGTCCTC  ATAGCTGGCAGTACTGTCCCGTTTTAGAGCTAGAAATAGCAAGTTA  ATCAGCCCCGTGCCATTTCTGTTTTAGAGCTAGAAATAGCAAGTTA  TCGAATTCATGGAGCAGCAGTTGTCCTC^e^  the same as above  the same as above  TCGAATTCATGGAGCAGCAGTTGTCCTC^e^  TCGAATTCATGGAGCAGCAGTTGTCCTC^e^  CCGGAATTCCACCACCCCACGCTCTTG^e^  CGGGGTACCATGGAGCAGCAGTTGTCCGCGCCGTCGGCGTCGCAGCGTGGCGG^b^  CTACACCATGGCGCCCAAGGTCGTCCACGC  GCAGCAGCACGCGCCCGCCGCCATCGAGCA  GAGCATCCTGGCGCCGGTGCCGGGGACAGT  GAGCTTCTTCGCCCCGCCGTCGTTGATCGCCCCCGTGCCATTTCTTGGT  GCCGTCGACGGCGCCGGCTCCAATGGGCGGCAGCGCC  CATTTCTTGGTGCAACGACTAC  TATGGTGCTATCCGCTTCGAT  GGGCAACCCAGTGACCAA  CCCTGCCGAATACGCCTAA  TGTATGCCAGTGGTCGTACCA | GGTACCTTACTCGGATATATTCATT^b^  ACGAGCTCGGATCCTGCAGGAGGAGATAAATGC^d^  AAGAATTCTTACTCGGATATATTCATT^e^  TCGTCGACTTAGTAGGGAGGATCCGGA^f^  CTTCCGGATGGCAACTTCCT^g^  CATACGCTGTCATGAGGCCAGTCT^g^  TCGGATCCTTAGAACAAGGGGCTGAGCT^h^  CAGGAGTTCATGACCGTCGTGCCACGGATCATCTGCACAAC  ACTTCTTGGATGCCCCCTTTGCCACGGATCATCTGCACAAC  TCGGATCCTTAGAACAAGGGGCTGAGCT^h^  CGCGGATCCTTACTTGGATGCCCCCTTTG^h^  CGCGGATCCTTAGTACGCCGGCATCATCAC^h^  CGCGGATCCTTAGTACGCCGGCATCATCAC^h^  the same as above  the same as above  CGCGGATCCTTAGAACAAGGGGGCGAGCTCCTGGATGGACTGCA^h^  GACGACGGCGCCGACCCCCTCGACGACGAC  ACCTTGGGCGCGTGCTCGTACACGATCACC  GTGTTCGGGGCGAAGACGAAGCCCGGCGCCAGCAGCATGCCGGCGGCC  CGACGGTCATGAACTCCT  TCGGATCCTTAATTTTGGTGGTGGTAGT  GGGACAGTACTGCCAGCTATGCCACGGATCATCTGCACAAC  AGAAATGGCACGGGGCTGATGCCACGGATCATCTGCACAAC  TCGGATCCTTAATTTTGGTGGTGGTAGT^h^  CGCGGATCCTTACTGCTGCACCGGCTCCTG^h^  CGCGGATCCTTACAAGAGCGTGGGGTGGTG^h^  CGCGGATCCTTACAAGAGCGTGGGGTGGTG^h^  TCGGATCCTTAATTTTGGTGGTGGTAGT^h^  TCGGATCCTTAATTTTGGTGGTGGTAGT^h^  the same as above  CGACCTTGGGCGCCATGGTGTAGATGATGA  GCGGCGGGCGCGTGCTGCTGCTGGGGCCAA  GGCACCGGCGCCAGGATGCTCGGCAACGGT  TGGCACGGGGGCGATCAACGACGGCGGGGCGAAGAAGCTCGCCGGGATAG  CCCATTGGAGCCGGCGCCGTCGACGGCGCAGCAGAAG  TGCATGTTGAAGAGATCCCAATA  CGAGAACCGAGCTCTCTTCAA  CGATTGCCTCGTCGGTCTT  CTCAAACGCCACGAGAATTTG  CCAGCAAGGTCGAGACGAA | Constructing overexpression vector  Constructing RNA interference vector  Constructing yeast two hybrid (Y2H) vector and protein expression vector  Constructing Y2H vector and protein expression vector  Constructing the kinase-inactive OsMPK4  Constructing the constitutively active OsMPK4  Constructing overexpression and protein expression vector  Amplifying *OsVQ14*  CRISPR targeting site 1  Amplifying OsVQ14  CRISPR targeting site 2  Constructing Y2H vector and detecting positive transgenic plants of *OsVQ14*-KO  Constructing OsVQ14-dC1 Y2H vector  Constructing OsVQ14-dC2 Y2H vector  Constructing OsVQ14-dNC Y2H vector  Constructing OsVQ14-dN1 Y2H vector  Constructing OsVQ14-dN2 Y2H vector  Constructing OsVQ14^1A^ protein expression vector  Constructing OsVQ14^2A^ protein expression vector  Constructing OsVQ14^3A^ protein expression vector  Constructing OsVQ14^5A^ protein expression vector  Analysing gene expression  Constructing overexpression and protein expression vector  Amplifying *OsVQ32*  CRISPR targeting site 1  Amplifying *OsVQ32*  CRISPR targeting site 2  Constructing Y2H vector  Constructing OsVQ32-dC1 Y2H vector  Constructing OsVQ32-dC2 Y2H vector  Constructing OsVQ32-dNC Y2H vector  Constructing OsVQ32-dN1 Y2H vector and detecting positive transgenic plants of *OsVQ32*-KO  Constructing OsVQ32-dN2 Y2H vector  Amplifying OsVQ32^1A^ protein expression vector  Amplifying OsVQ32^2A^ protein expression vector  Amplifying OsVQ32^3A^ protein expression vector  Amplifying OsVQ32^4A^ protein expression vector  Amplifying OsVQ32^6A^ protein expression vector  Amplifying OsVQ32^8A^ protein expression vector  Analysing gene expression  Analysing *ICS1* expression  Analysing *PAL1* expression  Analysing *PR10* expression  Analysing *Actin* expression |

^a^RGAP (Rice Genome Annotation Project: http://rice.plantbiology.msu.edu/) locus identifier.

^b^The underlined nucleotides are the digestion site of *Kpn*I.

^c^The underlined nucleotides are the digestion site of *Spe*I and *Kpn*I.

^d^The underlined nucleotides are the digestion site of *Sac*I and *Bam*HI.

^e^The underlined nucleotides are the digestion site of *Eco*RI.

^f^The underlined nucleotides are the digestion site of *Sal*I.

^g^The underlined nucleotides are the mutation sites.

^h^The underlined nucleotides are the digestion site of *Bam*HI.

**Table S3.** Rice *VQ* genes used in phylogenetic tree.

| Gene name | Gene ID | mRNA ID ^a^ | Protein length (aa) |
| --- | --- | --- | --- |
| *OsVQ1* | LOC_Os01g17050 | LOC_Os01g17050.1 | 236 |
| *OsVQ2* | LOC_Os01g46440 | LOC_Os01g46440.1 | 77 |
| *OsVQ3* | LOC_Os01g54400 | LOC_Os01g54400.1 | 232 |
| *OsVQ4* | LOC_Os01g59410 | LOC_Os01g59410.1 | 185 |
| *OsVQ5* | LOC_Os02g07690 | LOC_Os02g07690.1 | 230 |
| *OsVQ6* | LOC_Os02g15280 | LOC_Os02g15280.1 | 117 |
| *OsVQ7* | LOC_Os02g15290 | LOC_Os02g15290.1 | 127 |
| *OsVQ8* | LOC_Os02g33600 | LOC_Os02g33600.1 | 276 |
| *OsVQ9* | LOC_Os02g51740 | LOC_Os02g51740.1 | 311 |
| *OsVQ10* | LOC_Os03g20330 | LOC_Os03g20330.1 | 178 |
| *OsVQ11* | LOC_Os03g20440 | LOC_Os03g20440.1 | 216 |
| *OsVQ12* | LOC_Os03g26990 | LOC_Os03g26990.1 | 289 |
| *OsVQ13* | LOC_Os03g47280 | LOC_Os03g47280.1 | 144 |
| *OsVQ14* | LOC_Os03g57520 | LOC_Os03g57520.1 | 168 |
| *OsVQ15* | LOC_Os04g34050 | LOC_Os04g34050.1 | 300 |
| *OsVQ16* | LOC_Os04g55240 | LOC_Os04g55240.1 | 203 |
| *OsVQ17* | LOC_Os04g57030 | LOC_Os04g57030.1 | 152 |
| *OsVQ18* | LOC_Os05g12090 | LOC_Os05g12090.1 | 95 |
| *OsVQ19* | LOC_Os05g32460 | LOC_Os05g32460.1 | 222 |
| *OsVQ20* | LOC_Os05g41250 | LOC_Os05g41250.1 | 189 |
| *OsVQ21* | LOC_Os05g44270 | LOC_Os05g44270.1 | 271 |
| *OsVQ22* | LOC_Os06g33970 | LOC_Os06g33970.1 | 145 |
| *OsVQ23* | LOC_Os06g40090 | LOC_Os06g40090.1 | 205 |
| *OsVQ24* | LOC_Os06g41450 | LOC_Os06g41450.1 | 189 |
| *OsVQ25* | LOC_Os06g45570 | LOC_Os06g45570.1 | 214 |
| *OsVQ26* | LOC_Os07g06750 | LOC_Os07g06750.1 | 176 |
| *OsVQ27* | LOC_Os07g06760 | LOC_Os07g06760.1 | 188 |
| *OsVQ28* | LOC_Os07g06790 | LOC_Os07g06790.1 | 178 |
| *OsVQ29* | LOC_Os07g43140 | LOC_Os07g43140.1 | 253 |
| *OsVQ30* | LOC_Os07g48710 | LOC_Os07g48710.1 | 219 |
| *OsVQ31* | LOC_Os07g48800 | LOC_Os07g48800.1 | 138 |
| *OsVQ32* | LOC_Os08g01260 | LOC_Os08g01260.1 | 208 |
| *OsVQ33* | LOC_Os08g31660 | LOC_Os08g31660.1 | 427 |
| *OsVQ34* | LOC_Os09g20020 | LOC_Os09g20020.1 | 1138 |
| *OsVQ35* | LOC_Os09g20460 | LOC_Os09g20460.1 | 439 |
| *OsVQ36* | LOC_Os10g01240 | LOC_Os10g01240.1 | 97 |
| *OsVQ37* | LOC_Os11g03660 | LOC_Os11g03660.1 | 182 |
| *OsVQ38* | LOC_Os11g12790 | LOC_Os11g12790.1 | 165 |
| *OsVQ39* | LOC_Os12g03420 | LOC_Os12g03420.1 | 192 |
| *OsVQ40* | LOC_Os03g09045 | LOC_Os03g09045.1 | 249 |

a. The mRNA was selected if it encodes the longest protein of the gene.

**Table S4.** Arabidopsis *VQ* genes used in phylogenetic tree.

| Gene name | Gene ID | mRNA ID ^a^ | Protein length (aa) |
| --- | --- | --- | --- |
| *AtVQ1* | AT1G17147 | AT1G17147.1 | 98 |
| *AtVQ2* | AT1G21320 | AT1G21320.1 | 421 |
| *AtVQ3* | AT1G21326 | AT1G21326.1 | 239 |
| *AtVQ4* | AT1G28280 | AT1G28280.1 | 247 |
| *AtVQ5* | AT1G32585 | AT1G32585.1 | 220 |
| *AtVQ6* | AT1G32610 | AT1G32610.2 | 291 |
| *AtVQ7* | AT1G35830 | AT1G35830.1 | 302 |
| *AtVQ8* | AT1G68450 | AT1G68450.1 | 152 |
| *AtVQ9* | AT1G78310 | AT1G78310.1 | 311 |
| *AtVQ10* | AT1G78410 | AT1G78410.2 | 108 |
| *AtVQ11* | AT1G80450 | AT1G80450.1 | 177 |
| *AtVQ12* | AT2G22880 | AT2G22880.1 | 114 |
| *AtVQ13* | AT2G33780 | AT2G33780.1 | 204 |
| *AtVQ14* | AT2G35230 | AT2G35230.1 | 402 |
| *AtVQ15* | AT2G41010 | AT2G41010.1 | 238 |
| *AtVQ16* | AT2G41180 | AT2G41180.1 | 141 |
| *AtVQ17* | AT2G42140 | AT2G42140.1 | 172 |
| *AtVQ18* | AT2G44340 | AT2G44340.1 | 188 |
| *AtVQ19* | AT3G15300 | AT3G15300.1 | 219 |
| *AtVQ20* | AT3G18360 | AT3G18360.1 | 285 |
| *AtVQ21* | AT3G18690 | AT3G18690.1 | 222 |
| *AtVQ22* | AT3G22160 | AT3G22160.1 | 192 |
| *AtVQ23* | AT3G56710 | AT3G56710.2 | 151 |
| *AtVQ24* | AT3G56880 | AT3G56880.1 | 245 |
| *AtVQ25* | AT3G58000 | AT3G58000.1 | 175 |
| *AtVQ26* | AT3G60090 | AT3G60090.1 | 157 |
| *AtVQ27* | AT4G15120 | AT4G15120.1 | 193 |
| *AtVQ28* | AT4G20000 | AT4G20000.1 | 208 |
| *AtVQ29* | AT4G37710 | AT4G37710.2 | 123 |
| *AtVQ30* | AT4G39720 | AT4G39720.2 | 296 |
| *AtVQ31* | AT5G08480 | AT5G08480.2 | 173 |
| *AtVQ32* | AT5G46780 | AT5G46780.1 | 237 |
| *AtVQ33* | AT5G53830 | AT5G53830.1 | 243 |
| *AtVQ34* | AT5G65170 | AT5G65170.1 | 362 |

a. The mRNA was selected if it encodes the longest protein of the gene.
